# Unique Gene Expression and MR *T_2_* Relaxometry Patterns Define Chronic Murine Dextran Sodium Sulphate Colitis as a Model for Connective Tissue Changes in Human Crohn’s Disease

**DOI:** 10.1371/journal.pone.0068876

**Published:** 2013-07-23

**Authors:** Christine Breynaert, Tom Dresselaers, Clémentine Perrier, Ingrid Arijs, Jonathan Cremer, Leentje Van Lommel, Kristel Van Steen, Marc Ferrante, Frans Schuit, Séverine Vermeire, Paul Rutgeerts, Uwe Himmelreich, Jan L. Ceuppens, Karel Geboes, Gert Van Assche

**Affiliations:** 1 Translational Research Center for Gastrointestinal Disorders (TARGID), Department of Clinical and Experimental Medicine, KU Leuven, Leuven, Belgium; 2 Laboratory of Clinical immunology, Department of Microbiology and Immunology, KU Leuven, Leuven, Belgium; 3 Biomedical MRI/MoSAIC, Department of Imaging and Pathology, KU Leuven, Leuven, Belgium; 4 Gene Expression Unit, Department of Cellular and Molecular Medicine, KU Leuven, Leuven, Belgium; 5 Montefiore Institute, System and Modeling Unit, University of Liège, Liège, Belgium; 6 Translational Cell and Tissue Research, Department of Imaging and Pathology, KU Leuven, Leuven, Belgium; Charité-University Medicine Berlin, Germany

## Abstract

**Introduction:**

Chronically relapsing inflammation, tissue remodeling and fibrosis are hallmarks of inflammatory bowel diseases. The aim of this study was to investigate changes in connective tissue in a chronic murine model resulting from repeated cycles of dextran sodium sulphate (DSS) ingestion, to mimic the relapsing nature of the human disease.

**Materials and Methods:**

C57BL/6 mice were exposed to DSS in drinking water for 1 week, followed by a recovery phase of 2 weeks. This cycle of exposure was repeated for up to 3 times (9 weeks in total). Colonic inflammation, fibrosis, extracellular matrix proteins and colonic gene expression were studied. *In vivo* MRI *T*_2_ relaxometry was studied as a potential non-invasive imaging tool to evaluate bowel wall inflammation and fibrosis.

**Results:**

Repeated cycles of DSS resulted in a relapsing and remitting disease course, which induced a chronic segmental, transmural colitis after 2 and 3 cycles of DSS with clear induction of fibrosis and remodeling of the muscular layer. Tenascin expression mirrored its expression in Crohn’s colitis. Microarray data identified a gene expression profile different in chronic colitis from that in acute colitis. Additional recovery was associated with upregulation of unique genes, in particular keratins, pointing to activation of molecular pathways for healing and repair. *In vivo* MRI *T_2_* relaxometry of the colon showed a clear shift towards higher *T_2_* values in the acute stage and a gradual regression of *T_2_* values with increasing cycles of DSS.

**Conclusions:**

Repeated cycles of DSS exposure induce fibrosis and connective tissue changes with typical features, as occurring in Crohn’s disease. Colonic gene expression analysis revealed unique expression profiles in chronic colitis compared to acute colitis and after additional recovery, pointing to potential new targets to intervene with the induction of fibrosis. *In vivo T_2_* relaxometry is a promising non-invasive assessment of inflammation and fibrosis.

## Introduction

The chronic inflammatory bowel diseases (IBD), Crohn’s disease (CD) and ulcerative colitis (UC), are heterogeneous idiopathic inflammatory disorders of the intestine with a relapsing-remitting clinical course. The etiology remains unclear, but overall, an inappropriate immunologic response to commensal bacteria of the gut in genetically susceptible subjects is considered to be involved [1], [2]. In CD, which is in essence a transmural disease, chronic mucosal inflammation induces remodeling of the entire intestinal wall. This process is a cascade of events that includes epithelial cell damage and repair, angiogenesis and lymphangiogenesis and activation of immune cells and mesenchymal cells.

Mesenchymal cells, which are the major source of extracellular matrix (ECM) proteins, include (myo-) fibroblasts and smooth muscle cells of the muscularis mucosae and muscularis propria. Relapsing transmural inflammation in CD results in transmural lymphoid hyperplasia and in the accumulation of excess ECM proteins, including collagens. Intestinal strictures in CD are characterized by an increase in type V collagen, a collagen type produced in relatively large amounts by smooth muscle cells [3]. Collagens type IV and V are increased in the muscularis propria and around ganglia, while collagen type III is extensively present in ulcerations [4]. Also tenascin, a component of the ECM and synthesized mainly by fibroblasts, smooth muscle cells and myofibroblasts, is highly increased in active CD and UC [5]. In addition to these ECM changes, accumulation of myofibroblasts and alterations of the nerves induce fibromuscular obliteration of the submucosa, associated with thickening of the muscularis propria which results in a disturbed motility [6]. These events are the principal features in the genesis of the long-term complications of IBD such as strictures and perforating ulcers [2]. Neuronal and vascular changes make up the remaining connective tissue changes: these constitute a distinctive feature, and are even specific for CD [7].

Most if not all experimental animal models used to study the pathogenesis of IBD are acute or chronic without relapse and fail to reflect accurately the chronically relapsing inflammation that underlies the complications of human CD. Furthermore, recent evidence suggests that the pathways driving the inflammatory reaction in chronic murine colitis and in human CD may differ from those at onset of disease [8], [9]. A validated animal model of chronically relapsing inflammation is currently not available and would undoubtedly improve the understanding of the biology of remodeling and fibrosis of the bowel wall and assist the development of anti-fibrotic treatments [10], [11]. Multiple cycles of dextran sodium sulphate (DSS) induce chronic intestinal inflammation, but in spite of the technical ease, little attention has been given to the study of remodeling and fibrogenic responses to DSS [10], [12]–[14].

Non-invasive imaging tools that can assess connective tissue changes and can be applied repetitively, would be a major asset for these models, especially for the study of treatment efficacy. Ideally, these techniques should assess the full transmural extent of the disease and discern inflammatory from fibrotic lesions. The value of magnetic resonance imaging (MRI) as non-invasive assessment tools of transmural inflammation and extraluminal complications is increasingly recognized in human CD [15]. However, MRI data in murine ileitis or colitis are very limited [16]–[19].

Therefore, our aims were to investigate the connective tissue changes in a murine model of IBD with repeated cycles of DSS (1 week DSS followed by 2 weeks recovery; repeated up to 3 times) to mimic the relapsing nature of the disease. Colonic inflammation, fibrosis, extracellular matrix proteins and colonic gene expression were studied. Follow-up with *in vivo* MRI *T*_2_ relaxometry was used to investigate the value of this modality as a non-invasive assessment tool of bowel wall inflammation and fibrosis.

## Materials and Methods

### Mice

Female 6-weeks old C57BL/6 mice, obtained from Harlan (Horst, The Netherlands), were maintained in the Animal Care Facility of the Faculty of Medicine, University of Leuven (Belgium). Studies were approved by the local ethics committee for animal experimentation of the University of Leuven (P024–2010 and P134–2010).

### Induction and Evaluation of DSS Colitis

**Figure 1:**
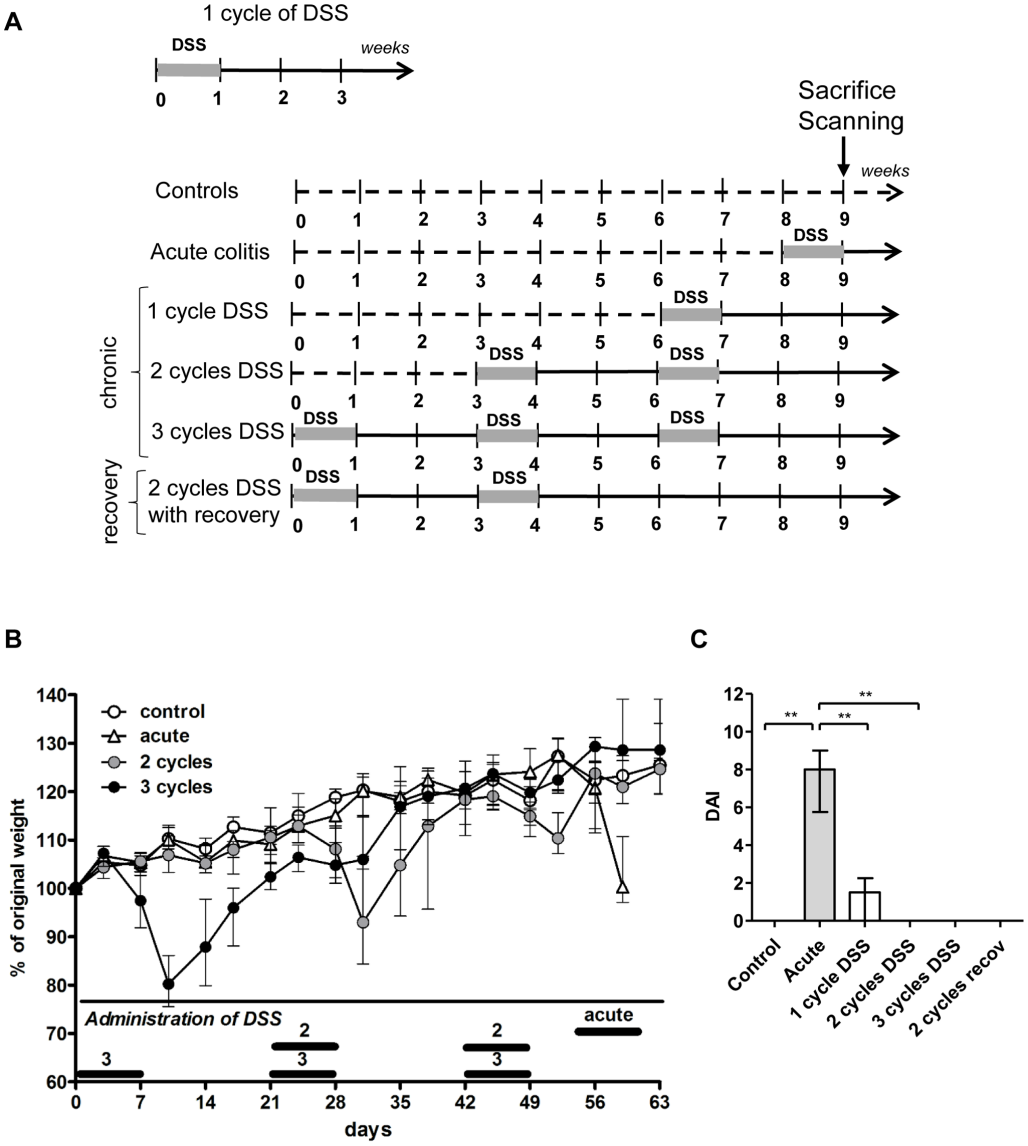
Experimental design, weight curves and disease activity index. (A) One cycle of DSS comprises 7 days of DSS followed by a recovery period of 14 days with normal drinking water. The study set-up consists of 6 different groups (n = 10 for each group with exception of the control group (n = 7) and 3-cycles group (n = 9). Acute colitis mice (n = 10) received 7 days of DSS without a recovery period prior to sacrifice and/or scanning. In a second group (n = 10) colitis was induced by DSS for 7 days followed by a recovery period of two weeks with normal drinking water, and this was defined as one cycle of DSS. In a third and fourth group, the DSS cycle was repeated twice (n = 10) or three times (n = 10). A fifth group (n = 10) received two cycles of DSS followed by an additional recovery period of three weeks with normal drinking water. Control mice (n = 7) received normal drinking water throughout. All mice were 6-weeks old at time of start of the 9-weeks study period and in this way age-matched at the time of sacrifice and scanning (15-weeks old). (B) Weight curves of control mice, acute, 2-, and 3-cycles mice. Data are expressed as medians with interquartile range (IQR). (C) The disease activity index (DAI) was determined at the end of the 9-weeks study period, based on body weight loss (one point for each 5% loss of weight), stool consistency (0, normal; 2, formed but very soft; 4, liquid) and presence of gross blood in the stools (0, none; 1, present). Data are expressed as medians with IQR. Mann-Whitney U testing (*p<0.05, **p≤0.01, ***p≤0.001).

1.5–2.0% DSS (35–50 000 kDa; MP Biomedicals, Illkirch, France) was added to drinking water during 7 days to induce colitis. Acute colitis mice (n = 10) received 7 days of DSS without a recovery period prior to sacrifice and/or scanning. In a second group (n = 10) colitis was induced by DSS for 7 days followed by a recovery period of two weeks with normal drinking water, and this was defined as one cycle of DSS (figure 1A). In a third and fourth group, the DSS cycle was repeated twice (n = 10) or three times (n = 10). A fifth group (n = 10) received two cycles of DSS followed by an additional recovery period of three weeks with normal drinking water. Control mice (n = 7) received normal drinking water throughout. Previously, we performed these experiments several times in different set-ups. These data showed important weight loss after the first cycle of DSS exposure but limited changes in weight after subsequent administrations of DSS. Therefore, we used a higher percentage of DSS (2.0%) during the second and third cycle compared to the 1.5% for the first administration. The data presented are the final experiment in which we combined all groups. Mice were weighted twice weekly. All mice were age-matched at the time of sacrifice and/or scanning. Animals were euthanized with sodium pentobarbital (Nembutal, Ovation Pharmaceuticals Inc. Deerfield, US). The disease activity index (DAI) was determined based on body weight loss (one point for each 5% loss of weight), stool consistency (0, normal; 2, formed but very soft; 4, liquid) and presence of gross blood in the stools (0, none; 1, present).

### Evaluation of Colonic Inflammation and Histology

The entire colon was removed, cleaned, weighted and measured from the ileocaecal junction to anus. A macroscopic damage score was calculated as the sum of the presence of adhesions (0, none; 1, mild to moderate; 2, moderate to severe), hyperemia (0, none; 1, present) and extent of colonic infiltration (one point for each centimeter of moderate inflammation or two points for each centimeter of severe inflammation evaluated by the thickness of the colon). One part of the most infiltrated distal colon was fixed in 4% formalin for histology and one part snap frozen. Hydroxyprolin assay was performed as previously described [20]. Wet and dry weight of a piece of the most infiltrated distal colon were determined by weighing tissue before and after drying (>18 hours at 60°C). Difference in weight was considered to be the water content. Histology was performed in paraffin embedded, 5 µm-thick longitudinal and transverse sections stained with hematoxylin and eosin. The microscopic score of inflammation was calculated as previously described [21]. The histological active disease score comprised the sum of neutrophil infiltration and epithelial defects, expressing the activity of disease and the potential for acute tissue damage during the last 24 hours prior to tissue sampling. Slides were scored by an experienced pathologist (K.G.) blinded to the experimental condition.

### Evaluation of Fibrosis and Chronic Wound Healing

Sections were stained using a picro-sirius red and Martius-scarlet-blue trichrome (MSB) staining highlighting connective tissue changes [22]. Images were acquired using a Zeiss Axiovert 200 microscope, a Zeiss Axiocam MRc5 camera and the Zeiss Axiovision 4.7.1.0 software imaging system. The surface of blue in mucosa and submucosa was quantified using ImageJ 1.45 s and used as an indication for the deposition of collagen [23]. The thickness of the mucosa and muscularis propria was calculated as mean value of two different points per mouse on uniform horizontal cross sections of colon crypts using ImageJ [23].

### Immunohistochemistry

Five µm-sections were deparaffinized and rehydrated. After antigen retrieval and blockade of endogenous peroxidases followed by washing steps, sections were incubated with primary antibodies for 1–2 hours at room temperature followed by incubation with the secondary antibodies during 45 minutes. After washing and amplification with Tyramide Cy3 (NEL704, Perkin Elmer), slides were incubated with DAPI (Sigma-Aldrich, cat# D9542), and finally mounted with Fluorsave reagent (Calbiochem cat# 345789). Following primary antibodies were used: polyclonal rabbit anti-α-smooth muscle actin (α-SMA) (Abcam ab5694) in a 1/100 dilution, polyclonal goat anti-vimentin (Sigma V4630) in a 1/2500 dilution, polyclonal rabbit anti-collagen I (Abcam ab21286) in a 1/250 dilution, monoclonal rat anti-tenascin C (Abcam ab6346) in a 1/4500 dilution and polyclonal rabbit anti-collagen III in a 1/500 dilution (ab7778). As secondary antibodies, we used donkey anti-rabbit Alexa 488 (Jackson 711-545-152) in a 1/400 dilution and donkey anti-goat HRP (Jackson 705-035-147) in a 1/500 dilution.

### Whole-transcript Expression Microarray Analysis

Total RNA was extracted from snap frozen colon (Qiagen RNeasy Mini Kit cat# 74106). Total RNA (200 ng) was used to analyze the mRNA expression via Mouse Gene 1.0 ST arrays according to manufacturer’s manual 4475209 Rev.B (Applied biosystems, CA) and 702808 Rev.6 (Affymetrix, CA). Briefly, in the first cycle, double stranded cDNA was prepared with random hexamers tagged with a T7 promoter sequence followed by the generation of cRNA using the GeneChip WT Synthesis and Amplification kit (Applied biosystems). cRNA concentration after cleanup was measured with the NanoDrop ND-1000 spectrophotometer (NanoDrop Technologies, DW). In the second cycle, sense oriented single-stranded DNA containing dUTP is generated and the concentration is, after cleanup, measured using the nanodrop. The cRNA is hydrolysed and the single stranded DNA is fragmented using uracil DNA glycosylase (UDG) and apurinic/apyrimidinic endonuclease 1 (APE1) (GeneChip WT terminal Labeling kit, Affymetrix). The quality of fragmentation (fragments should be between 40 and 70 nucleotides) is checked on the bioanalyzer (Agilent, Waldbronn, Germany). The fragmented DNA is labeled by terminal deoxynucleotidyl transferase (TDT) with the Affymetrix DNA Labeling reagent that is covalently linked to biotin (GeneChip WT terminal Labeling kit, Affymetrix). Labeled DNA was hybridized to the array during 17 h at 45°C. The arrays were washed and stained in a fluidics station using the GeneChip hybridization, Wash end Stain kit (Affymetrix) and scanned using the Affymetrix 3000 GeneScanner. For data analysis, all image files were generated using the Affymetrix GeneChip command console (AGCC). The raw data were analyzed with RMA sketch using the standard settings for Gene 1.0 ST arrays of Expression Console in the AGCC software (Affymetrix). The microarray data were deposited at Gene Expression Omnibus under the series accession number GSE42768 (http://www.ncbi.nlm.nih.gov/geo/query/acc.cgi?token=ztwzpaseamwoora&acc=GSE42768), and were handled in accordance with the MIAME (Minimum Information About a Microarray Experiment) guidelines. The microarray data were analysed using Bioconductor tools in R (version 2.12.2, http://www.r-project.org) [24]. Probe level analysis was performed on the Affymetrix raw data (.cel files) with the robust multichip average method to obtain a log2 expression value for each gene probe set [25]. Unsupervised complete-linkage hierarchical clustering, using Euclidian distance as metric, was performed to visualize gene (probe set)/sample relationship. The clustering results were visualized as a 2-dimensional heatmap with 2 dendograms, one indicating the similarity between samples and the other indicating the similarity between gene probe sets. For comparative analysis, linear models for microarray data (LIMMA) [26] was performed for all the gene probe sets (n = 35511) present on the microarray to identify probe sets that are different between the groups, based on moderated *t*-statistics. The p-values derived from the moderated *t*-statistics were adjusted for multiple testing using the Benjamini and Hochberg false discovery rate (FDR) method [27]. The Bio Functional Analysis tool in the Ingenuity Pathway Analysis program (Ingenuity Systems®, www.ingenuity.com) was used to identify biological functions that were most significant to the datasets of differentially expressed gene probe sets, identified by LIMMA analyses.

### MRI - *T_2_* Relaxometry

Six mice per experimental condition (control mice: only 5) underwent MRI before sacrifice. Mice were anesthetized with 1.5–2% isoflurane mixed with air during the procedure. No paralytic agents were used. Images were acquired with a 9.4 T BioSpec system (Bruker Biospin, Ettlingen, Germany; horizontal bore, 20 cm) equipped with an actively shielded gradient insert (1200 mT/m) and using a 3.5 cm volume resonator (Rapid Biomedical, Rimpar, Germany). For planning purposes 2D respiration-triggered sagittal and coronal fat suppressed *T_2_*-weighted (*T_2_*w) images were recorded (RARE; TEeff = 52 ms, TR = 4800 ms, FOV = 3×3 cm, matrix = 256×256; slice thickness = 1 mm). *T_2_* maps were recorded from the same locations (multi-slice-multi-echo sequence, TR = 4000 ms, TE = 10–100 ms, 4 dummy scans, 3 averages, matrix 128×128, fat suppression, other parameters identical) covering the distal part of the colon. Maps were calculated using a plug-in for ImageJ. [23], [28] Regions of interest delineating the colon wall were manually drawn on the *T_2_*w images, acquired with the same geometry as the *T_2_* map, and then copied to the *T_2_* map. Histograms were created from the *T_2_* maps from three slices over the distal part of the colon. The final histogram per condition was calculated as a mean value of the sum of individual histograms per mouse and corrected for the total number of observed pixels per mouse.

### Statistical Analysis

Statistical analysis and calculations were performed using GraphPad Prism 5.03 (GraphPad, La Jolla, CA, USA) and R (version 2.7.2, http://www.r-project.org). Data are represented as medians (25% percentile, 75% percentile) and the individual p-values for two groups were obtained using Mann-Whitney U testing (*p<0.05, **p≤0.01, ***p≤0.001), unless annotated differently. Differences were considered statistically significant at p<0.05. For the statistical analysis of the MRI *T_2_* profiles, *T_2_* maps were analyzed by first carrying out a variable selection from random forests using both backwards variable elimination (for the selection of small sets of non-redundant variables) and selection based on the importance spectrum (varSelRF package in R). On the basis of the selected *T_2_* values, a non-parametric MANOVA was subsequently performed to compare the groups of interest on the joint set of retained *T_2_* values. For the analysis of the histograms, a Bonferroni correction was used to correct for multiple testing.

## Results

### Repeated Cycles of DSS Induce Relapse and Remission

The experimental design is summarized in Figure 1A. First, we studied the effect of 2 cycles of DSS and compared this to acute colitis (DSS for 7 days without recovery period). To study the effect of one cycle less or more, we added a 1-cycle group and a 3-cycles group. In an additional group, we examined the effect of an additional recovery period of three weeks after 2 cycles of DSS administration. During the first week of exposure to DSS, mice showed about 20% body weight loss and had bloody stools. This was followed by a complete recovery during the next 2 weeks. The following cycles of DSS induced less weight loss (Figure 1B). A disease activity index (DAI) was calculated on the basis of body weight loss, stool consistency and presence of gross blood in the stools at time of sacrifice. The highest DAI (DAI = 8.0 (5.8–9.0) was observed after the first week of DSS exposure due to weight loss and bloody stools. At the end of the following 2-weeks recovery, the DAI decreased to 1.5 (0–2.3). The DAI further decreased to 0 at the end of 2 and 3 cycles of DSS (Figure 1C). Only one mouse died during the experiment, i.e. after the first administration of DSS.

### Chronic DSS Colitis is Associated with Thickening and Infiltration of the Colon

**Figure 2:**
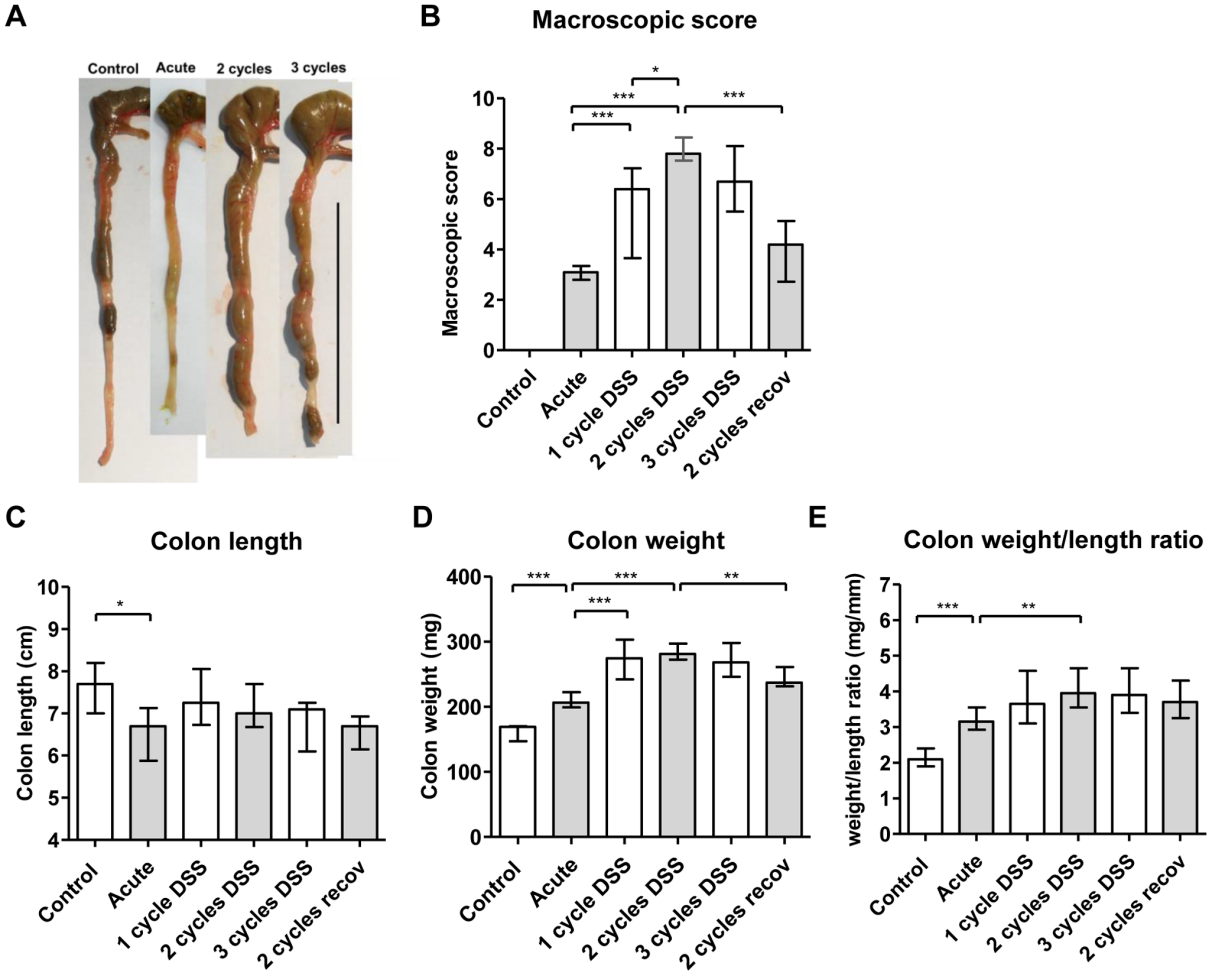
Inflammation of the colon. (A) Representative pictures of the colon in control mice, acute, 2-, and 3-cycles DSS colitis mice. The black bar represents 5 cm. (B) Macroscopic damage score of the colon in the 6 groups. (C) Length of colon shortened significant after 7 days of DSS (acute DSS colitis) compared to the control mice. (D) Weight of colon after removal of the feces. (E) Weight/length ratio of the colon. All data are expressed as medians with IQR. Mann-Whitney U testing (*p<0.05, **p≤0.01, ***p≤0.001).

In contrast to the DAI, the macroscopic damage score increased during the 2-weeks recovery phase after one administration of DSS (p<0.001), and increased even more with one or two additional cycles of DSS (2 cycles vs. 1 cycle, p = 0.010). During prolonged recovery after the second cycle of DSS (2-cycles with prolonged recovery), there was however a significant decrease of the macroscopic score (Figure 2A,B). The colon length was reduced significantly after one week of DSS exposure compared to control colon (p = 0.040), but tended to normalize during the 2 weeks of recovery. After 2 or more cycles of DSS, colon lengths were similar to those of control mice (Figure 2C). A significant increase in colon weight was observed in the chronic conditions with a maximum after 2 cycles of DSS, leading to a significantly higher weight/length ratio compared to acute colitis (p = 0.006) (Figure 2D,E). During additional recovery of 3 weeks after the second cycle of DSS, there was a significant decrease in colon weight (Figure 2D).

### Repeated DSS Exposure Induces Typical Histological Features of Crohn’s Disease

**Figure 3:**
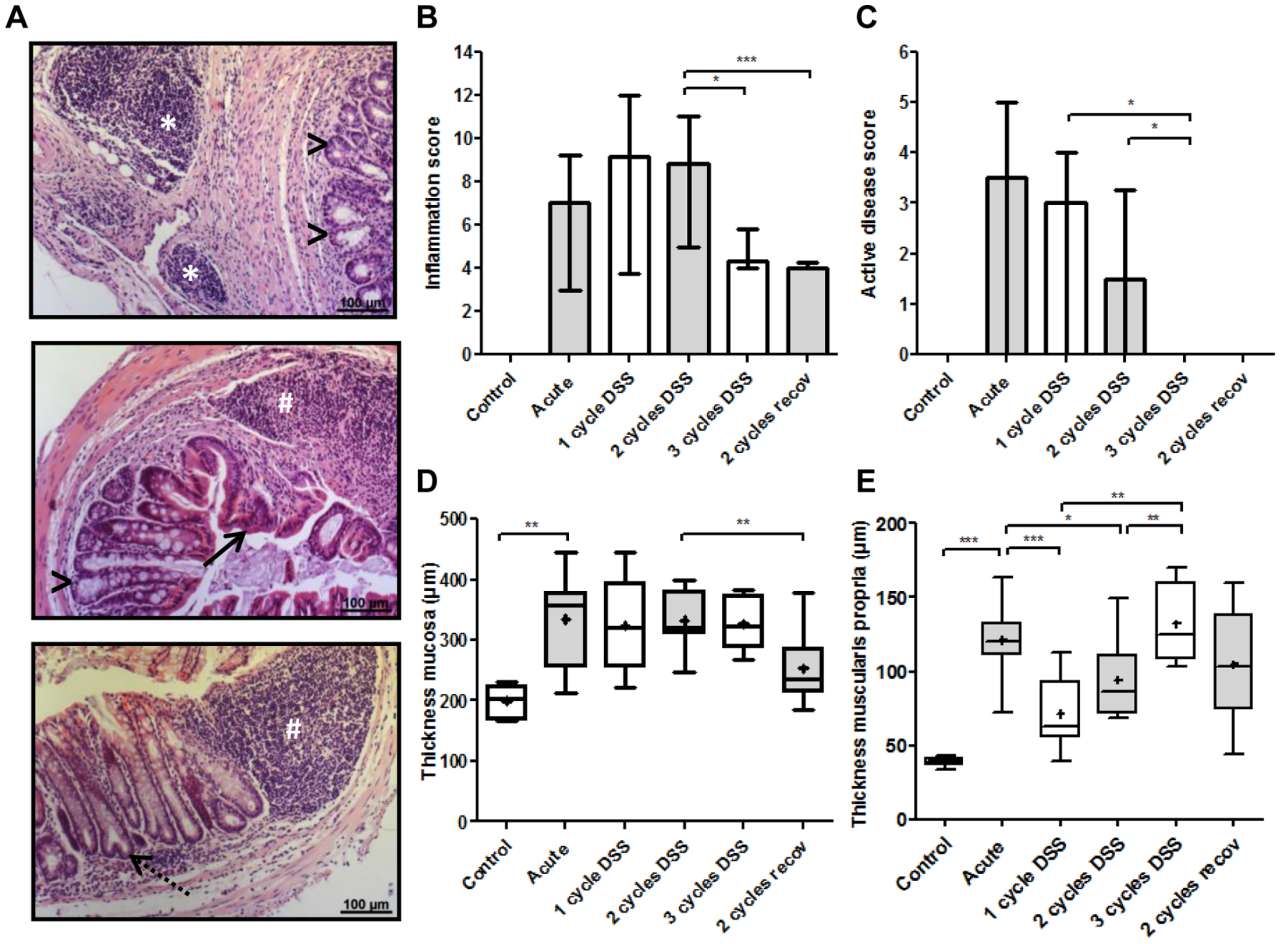
Histological read-outs of colitis. (A) Representative pictures of colon sections from mice after 2 and 3 cycles of DSS exposure. The repeated cycles of DSS induce persistent and dense infiltration of deeper layers with mononuclear cell aggregates in the subserosa (*), crypt dilatation (>), re-epithelialization (→) and crypt fission (dotted →). (B) Microscopic inflammation score. Three sections per animal were evaluated. The details of this score are shown in supplementary figure 1. Data are expressed as medians with IQR. (C) The lower degree of inflammation after 3 cycles and 2 cycles with prolonged recovery is associated with a lower histological active disease score. The histological active disease score comprised the sum of neutrophil infiltration and epithelial defects, expressing the activity of disease and the potential for acute tissue damage during the last 24 hours prior to tissue sampling. (D) Thickness of the mucosa and (E) thickness of the muscularis propria. Two sections per animal were evaluated. The mean is annotated as +. Data are expressed as box and whiskers and 5–95 percentile are plotted. Mann-Whitney U testing (*p<0.05, **p≤0.01, ***p≤0.001).

In the acute DSS colitis, typical histological inflammatory changes including infiltration of cells in mucosa and submucosa, loss of surface cells and re-epithelialization, crypt dilatation and crypt fissioning were found. After 2 and 3 cycles of DSS, these changes persisted throughout all layers, and we observed the appearance of lymphoid aggregates in the submucosa and subserosa, a unique and typical feature of CD (Figure 3A–B). The histological inflammation score was high after 1 week of DSS exposure and further increased in the 2-weeks recovery phase and during an additional cycle of DSS (Figure 3B). During a third cycle of DSS, architectural changes were less prominent, while mononuclear cell infiltration persisted but less neutrophils were present compared to the 2-cycles colon, leading to a lower histological inflammation score (Figure S1). This correlated with a lower histological active disease score (Figure 3C). This histological active disease score is expressing the activity of disease and the potential for acute tissue damage during the last 24 hours prior to tissue sampling. To differentiate between the effect of edema and remodeling, the thickness of the mucosa and muscularis propria was measured (Figure 3D–E). Repeated cycles of DSS induced persistent thickening of the mucosa, associated with ongoing inflammation. With more cycles of DSS, representing more chronic disease, a significant increase in thickness of the muscularis propria was observed. We also studied the effect of prolonged recovery after 2 cycles of DSS exposure on histological characteristics. The histological inflammation significantly decreased during prolonged recovery (p<0.001) (Figure 3B). Prolonged recovery also led to a normalization in mucosal thickness, expressing the plasticity of the mucosa to restore the architecture during a healing response. However, it was not associated with a recovery of the structural changes in the muscular layers, indicating persistent mesenchymal remodeling.

### Repeated DSS Exposure is Associated with More Connective Tissue Changes

**Figure 4:**
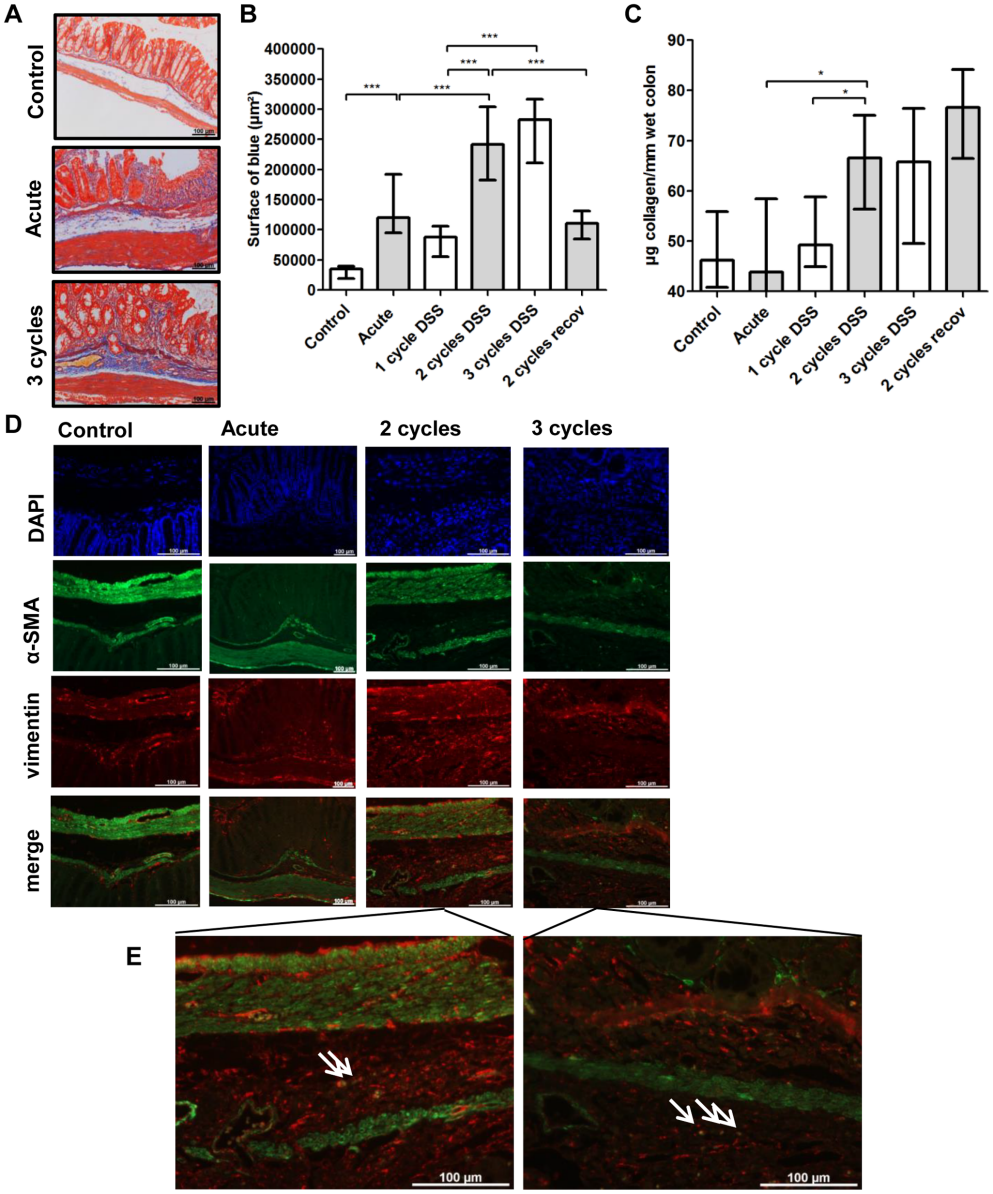
Fibrogenic changes in the colon. (A) Representative pictures of an Martius-scarlet-blue (MSB) staining of control mice, acute colitis and 3-cycles mice. A clear deposition of collagen in the submucosa is present in chronic DSS colitis. (B) Histological scoring of fibrosis using a MSB-staining shows that more cycles of DSS are associated with more fibrosis (surface of blue in mucosa and submucosa). More recovery is associated with a lower degree of fibrosis. (C) Hydroxyprolin assay. Data are expressed as µg collagen per mm wet colon. Data are expressed as medians with IQR. Mann-Whitney U testing (*p<0.05, **p≤0.01, ***p≤0.001). (D-E) Analysis of the mesenchymal cells in the submucosa shows the presence of myofibroblasts (α-SMA^+^ and vimentin^+^) in the submucosa after 2 and 3 cycles, indicated with the arrows. These cells are absent in control colon and in acute colitis.

Martius-scarlet-blue staining showed abundant collagen deposition in the mucosa and submucosa after 2 and 3 cycles of DSS exposure (Figure 4A). Analysis of the surface of blue in mucosa and submucosa revealed a higher fibrosis score after 2 and 3 cycles compared to 1 cycle of DSS (p<0.001) (Figure 4B). After prolonged recovery, there was a lower degree of fibrosis (2 cycles versus 2 cycles with additional recovery, p<0.001). These findings were confirmed using a hydroxyprolin assay (2 cycles versus acute and 1 cycle, p = 0.043 and p = 0.019 respectively) (Figure 4C). The 2-cycles with recovery group tended to have higher levels of collagen compared to the 2-, and 3-cycles group (p = 0.143 and p = 0.133 respectively).

**Figure 5:**
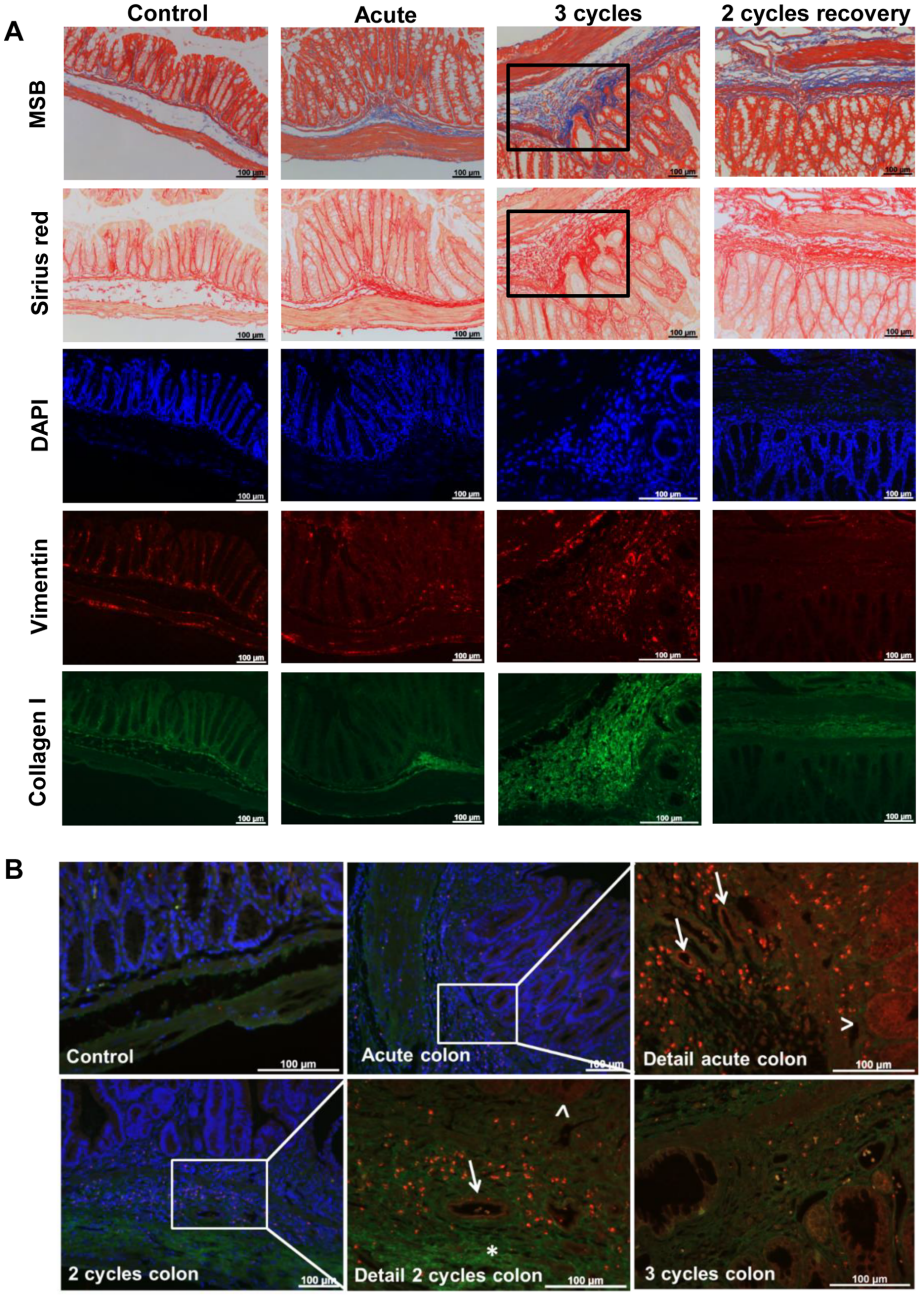
ECM changes in acute and chronic DSS colitis. (A) Representative pictures of the collagen I staining of control, acute, 3-cycles and 2-cycles with recovery colon and corresponding MSB and picro-sirius red staining. The rectangle in the upper pictures is shown with higher magnification in the lower pictures. (B) Representative pictures of the collagen III and tenascin staining in control, acute, 2-cycles and 3-cycles colon (merge image, blue = DAPI, green = collagen III and red = tenascin). The rectangle in the picture of the acute and 2-cycles colon is shown with higher magnification in the next picture (merge of collagen III and tenascin staining). No tenascin is observed in control colon. In the acute model, tenascin stains as a dot-like pattern in the mucosa and submucosa, indicating the tenascin-producing cells. Also a linear deposition of tenascin is seen around some crypts (>) and in blood vessels present in the submucosa (→). In the 2-cycles colon, the same expression of tenascin is observed. More collagen III is present in the submucosa (*). In the 3-cycles colon, less tenascin-positive cells are observed.

Myofibroblasts (vimentin and α-SMA positive) were present in the submucosa after 2 or more cycles and not in the acute phase (Figure 4D,E). The muscularis propria was clearly thicker (Figure 3E) and more infiltrated with vimentin positive cells after 2 and 3 cycles compared to acute colitis (Figure 4E). Deposition of collagen I, correlating well with the MSB and picro-sirius red staining, was present in regions in the submucosa around vimentin positive cells, i.e. myofibroblasts, after 2 or 3 cycles (Figure 5A). Collagen III, the newly synthesized collagen observed in wound healing response, was clearly present in the submucosa in acute colitis, and even more so after 2 and 3 cycles, when collagen III infiltration between the smooth muscle cells of the muscularis propria was observed (Figure 5B). With additional recovery after 2 cycles, less collagen III was present (data not shown).

As in the human colon, tenascin staining was negative in control colon (Figure 5B). In the acute phase, tenascin was clearly present in the mucosa and submucosa in individual cells. A linear deposition of tenascin around the crypts was also seen, as expected in acute colitis. The mucosal linear deposition and submucosal spots persisted after 2 cycles, correlating with persisting tissue damage. The expression of tenascin decreased after 3 cycles and with prolonged recovery after the second cycle, in association with restoration of tissue damage.

### Distinct Gene Expression Clusters Associated with Induction of Fibrosis and with Additional Recovery

**Figure 6:**
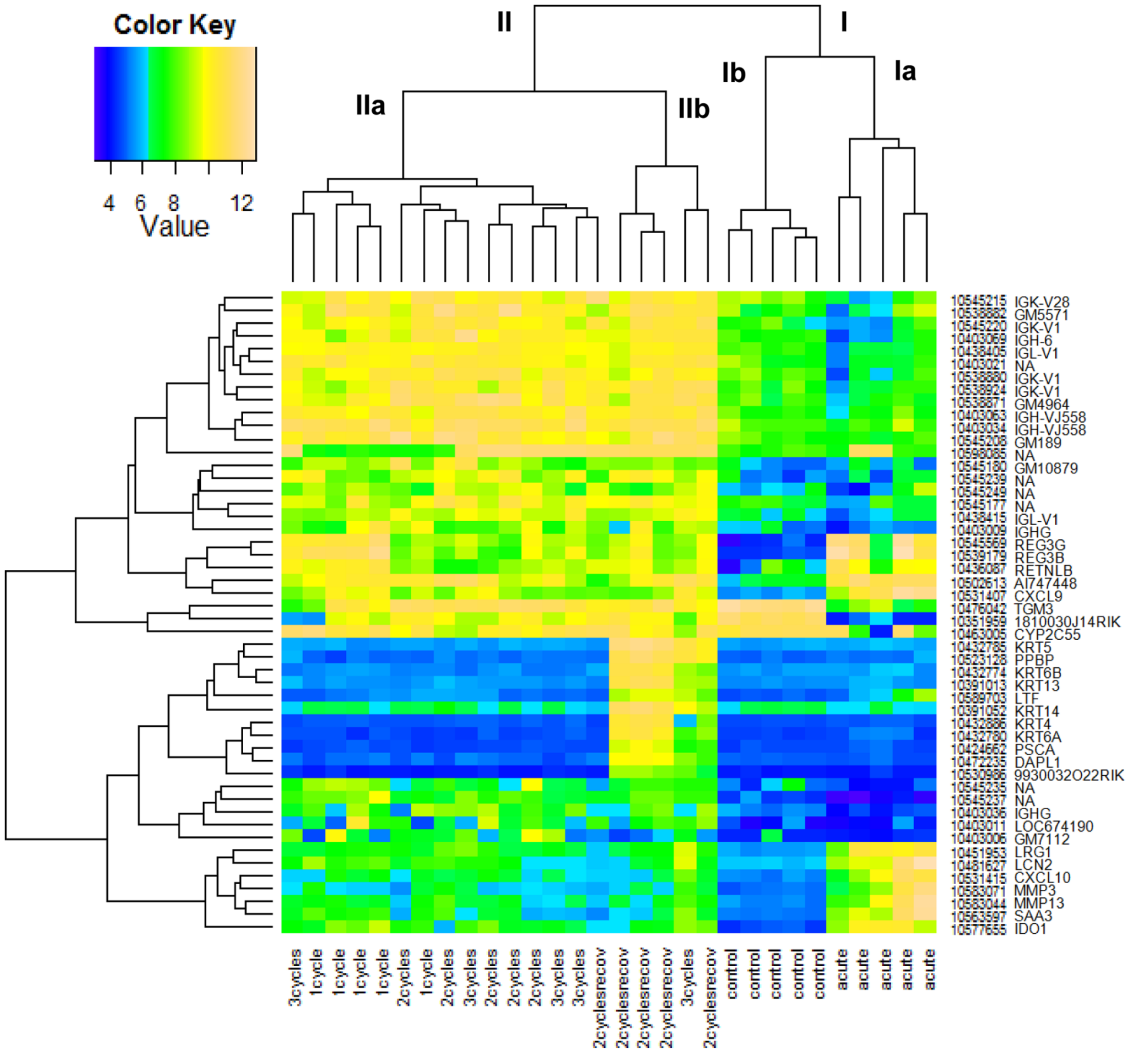
Unsupervised hierarchical cluster analysis based on log2 expression values of top 50 most variable gene probe sets. Individual samples are shown in columns and gene probe sets in rows. The log2 expression values for individual genes are indicated by color, as shown in the scale (color key), with yellow indicating a high level of expression and blue a low level of expression. The abbreviations of the individual genes and gene probe IDs are explained in Table S1.

To study the differences in colonic gene expression levels between acute and more chronic colitis, we performed a micro-array on colonic tissue of 5 animals per group, followed by an unsupervised hierarchical clustering of the top 50 gene probe sets with the highest variation in expression across the 30 arrays (Figure 6, Table S1). This allowed us to clearly identify distinct gene clusters related to the different conditions. Two major clusters were observed. Cluster I comprised two subclusters: the control group and the acute DSS colitis group. Cluster II contained also two subclusters: IIa included the samples of chronic colitis, so after 1, 2 and 3 cycles DSS, and IIb mainly the samples after 2 cycles with prolonged recovery. So, even in an unsupervised analysis, colonic gene expression showed clear differences between chronic and acute colitis, and between chronic colitis and prolonged recovery after the second cycle of DSS exposure.

**Figure 7:**
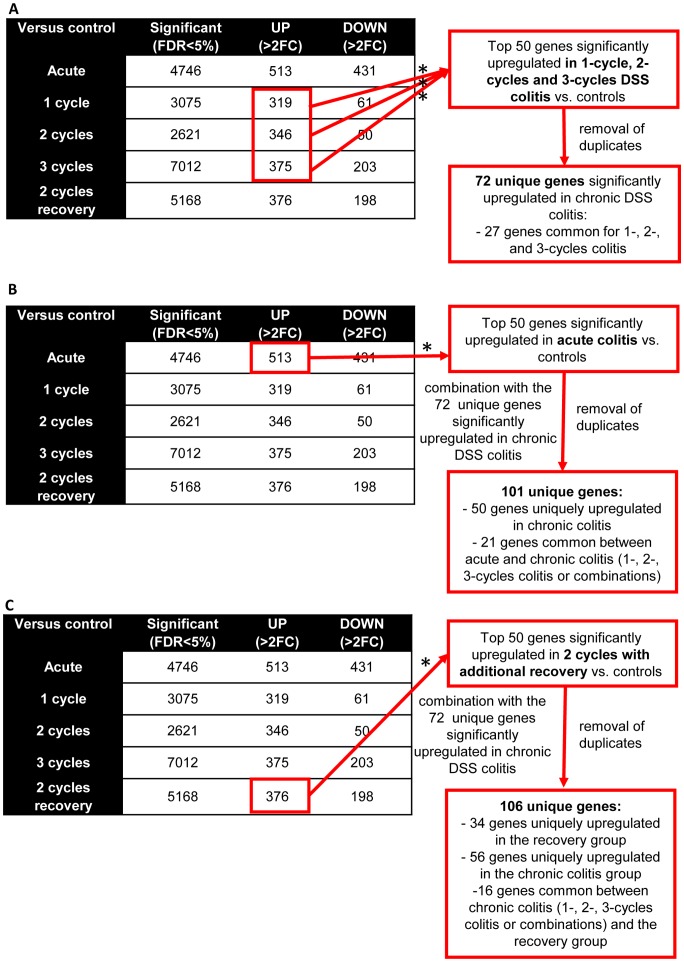
Identification of the comparisons between the differentially expressed probe sets of the different groups. (A) Identification of the top 50 significantly upregulated genes of 1-, 2-, and 3-cycles DSS colitis (FDR<0.05, FC>2) (most upregulated compared to controls). The analysis of these genes is shown in Figure 8. (B) Identification of the top 50 significantly upregulated genes of acute colitis and made comparison with the genes identified in chronic colitis. The analysis of these genes is shown in Figure 9. (C) Identification of the top 50 significantly most upregulated genes (fold change compared to controls) after additional recovery. The analysis of these genes is shown in Figure 10A. (*after removal of duplicates, of gene probe sets with unmapped IDs and of genes coding for gammaglobulin chains).

**Figure 8:**
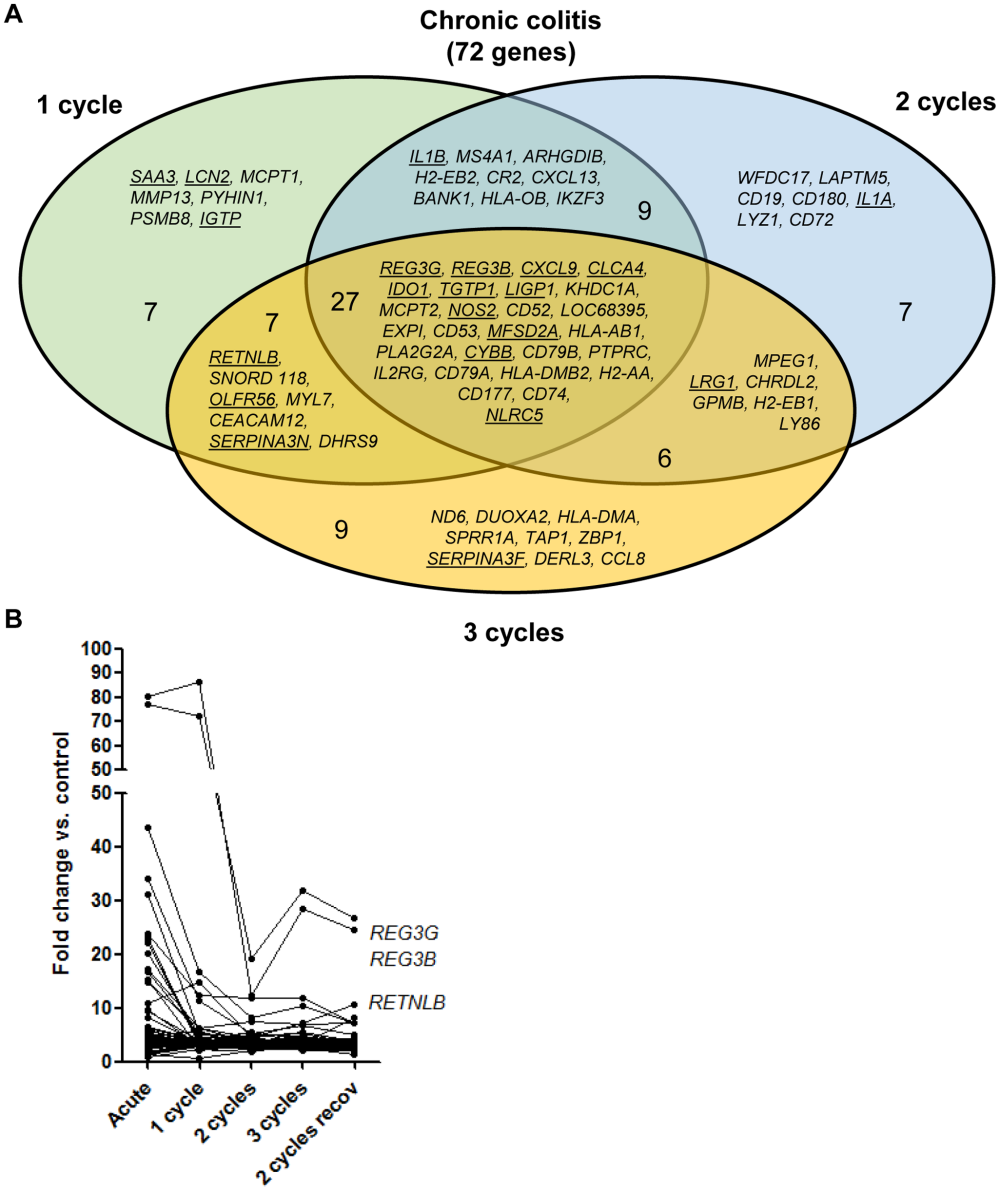
Colonic gene expression in chronic DSS colitis. (A) Venn diagram of the differentially expressed probe sets of 72 genes identified in the top 50 significantly upregulated genes of 1-, 2-, and 3-cycles DSS colitis (FDR<0.05, FC>2). Among these, 27 genes were commonly upregulated in 1-, 2-, and 3-cycles DSS colitis. The biological function of these 27 genes is summarized in Table 1. The abbreviations of the individual genes and gene probe IDs are explained in the Table S2. (B) Individual fold change expression of these 72 significantly upregulated genes vs. controls in chronic DSS colitis.

To study which genes are upregulated in chronic colitis, we looked at the top 50 genes significantly upregulated (FDR<0.05, FC>2) after 1, 2 and 3 cycles of DSS. The made comparisons are showed in Figure 7A. After removal of duplicates, of gene probe sets with unmapped IDs following the Ingenuity Pathway Analysis program, and of genes coding for gammaglobulin chains, 72 unique genes were identified out for 1, 2 and 3 cycles colitis combined (Figure 8A, Table S2). The kinetic changes of these 72 genes are shown in figure 8B. Of these 72 significantly upregulated genes, 27 were common for 1, 2 and 3 cycles colitis. This corresponds to the identified cluster of chronic colitis after unsupervised analysis. The biological functions of these 27 genes are summarized in Table 1.

**Table 1:** Biological function of selected genes in chronic and acute colitis and after additional recovery.

Physiological system development and function of the 27 commonly significantlyupregulated genes of the top 50 significantly upregulated genes in 1-, 2-, and3-cycles DSS colitis (fold change compared to controls) (FDR<0.05, FC>2)(cfr. Figure 8A)		P-value		# molecules
Hematological system development and function	5.96E-13–1.90E-02		17	
Immune cell trafficking	2.74E-12–1.90E-02		14	
Tissue morphology	1.32E-08–1.90E-02		12	
Hematopoiesis	3.12E-06–1.74E-02		9	
Humoral immune response	3.97E-06–9.53E-03		10	
**Physiological system development and function of the 30 uniquely upregulated** **genes of the top 50 significantly upregulated genes in acute DSS colitis** **(fold change compared to controls) vs. the 72 significantly upregulated genes in chronic** **colitis (cfr. ** **Figure 9A** **)**	**P-value**		**# molecules**	
Hematological system development and function	4.68E-14–7.96E-03		20	
Immune cell trafficking	4.68E-14–7.51E-03		15	
Tissue morphology	3.71E-13–6.49E-03		18	
Tissue development	2.84E-11–8.02E-03		14	
Cell-mediated immune response	7.51E-10–6.02E-03		5	
**Physiological system development and function of the 65 uniquely** **significantly upregulated genes in the 2-cycles with additional recovery group vs.** **2-cycles DSS colitis(cfr. ** **Figure 10B-C** **)**	**P-value**		**# molecules**	
Embryonic development	7.90E-11–4.75E-02		14	
Hair and skin development and function	7.90E-11–4.75E-02		16	
Organ development	7.90E-11–4.75E-02		15	
Organismal development	7.90E-11–4.75E-02		14	
Tissue development	7.90E-11–4.75E-02		20	

**Figure 9:**
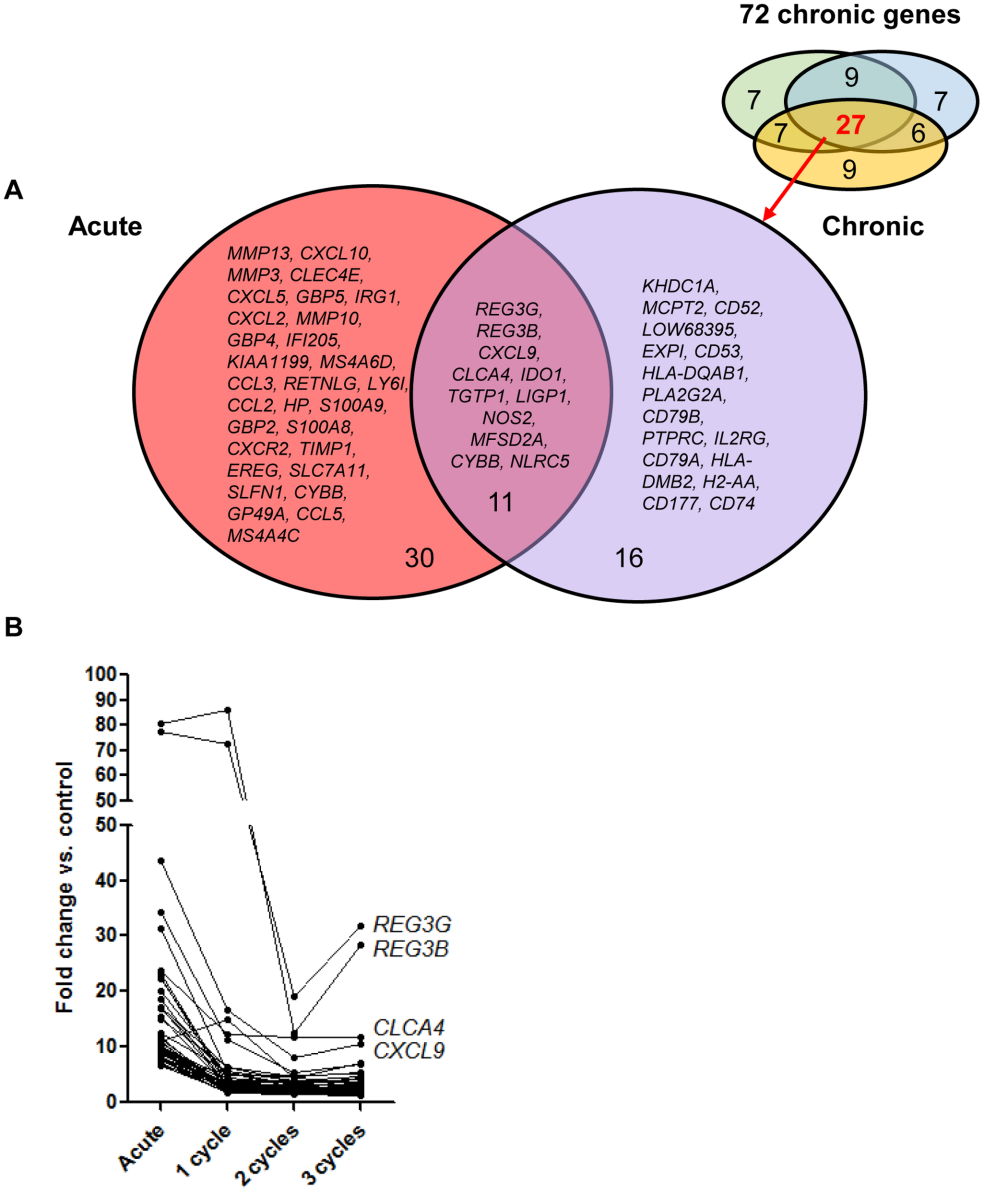
Colonic gene expression in acute DSS colitis. (A) Venn diagram of the differentially expressed probe sets of the top 50 significantly upregulated genes in acute DSS colitis (fold change compared to controls) (Table S3) (FDR<0.05, FC>2). These genes were compared with the 72 genes identified in chronic colitis. In total, 30 genes were uniquely upregulated in acute colitis. The biological functions of these 30 genes uniquely upregulated in acute colitis are summarized in Table 1. Out of these 50 genes, 21 genes were common in acute and chronic colitis (underlined in Figure 7). Only 11 out of these 21 genes were common with the 27 common upregulated genes in chronic colitis. For simplicity of the figure, only these 27 genes are shown in the venn diagram for the chronic condition. (B) Individual fold change expression of the top 50 most upregulated genes in acute DSS colitis vs. controls. The abbreviations of the individual genes and gene probe IDs are explained in Table S3.

To look for differences in colonic gene expression between acute and chronic colitis, we compared the 72 significantly upregulated genes in chronic colitis (Figure 8) with the top 50 significantly upregulated genes in acute colitis (Table S3). The made comparisons are showed in Figure 7B. In total, after removal of duplicates, of unmapped IDs and of genes coding for gammaglobulin chains, 101 unique genes could be identified. Fifty of these genes were uniquely upregulated in chronic colitis (1-, 2-, 3-cycles colitis or combinations) and only 21 genes were common between acute and chronic colitis (underlined in Figure 8). Eleven out of these 21 genes were common with the 27 common upregulated genes in chronic colitis (*REG3G, REG3B, CXCL9, CLCA4, IDO1, TGTP1, LIGP1, NOS2, MFSD2A, CYBB, NLRC5*). Thirty out of the 101 genes were uniquely upregulated in acute colitis (Figure 9A). The biological functions of these 30 genes uniquely upregulated in acute colitis are summarized in Table 1. The kinetic changes of upregulation of the top 50 significantly upregulated genes in acute colitis is shown in Figure 9B.

**Figure 10:**
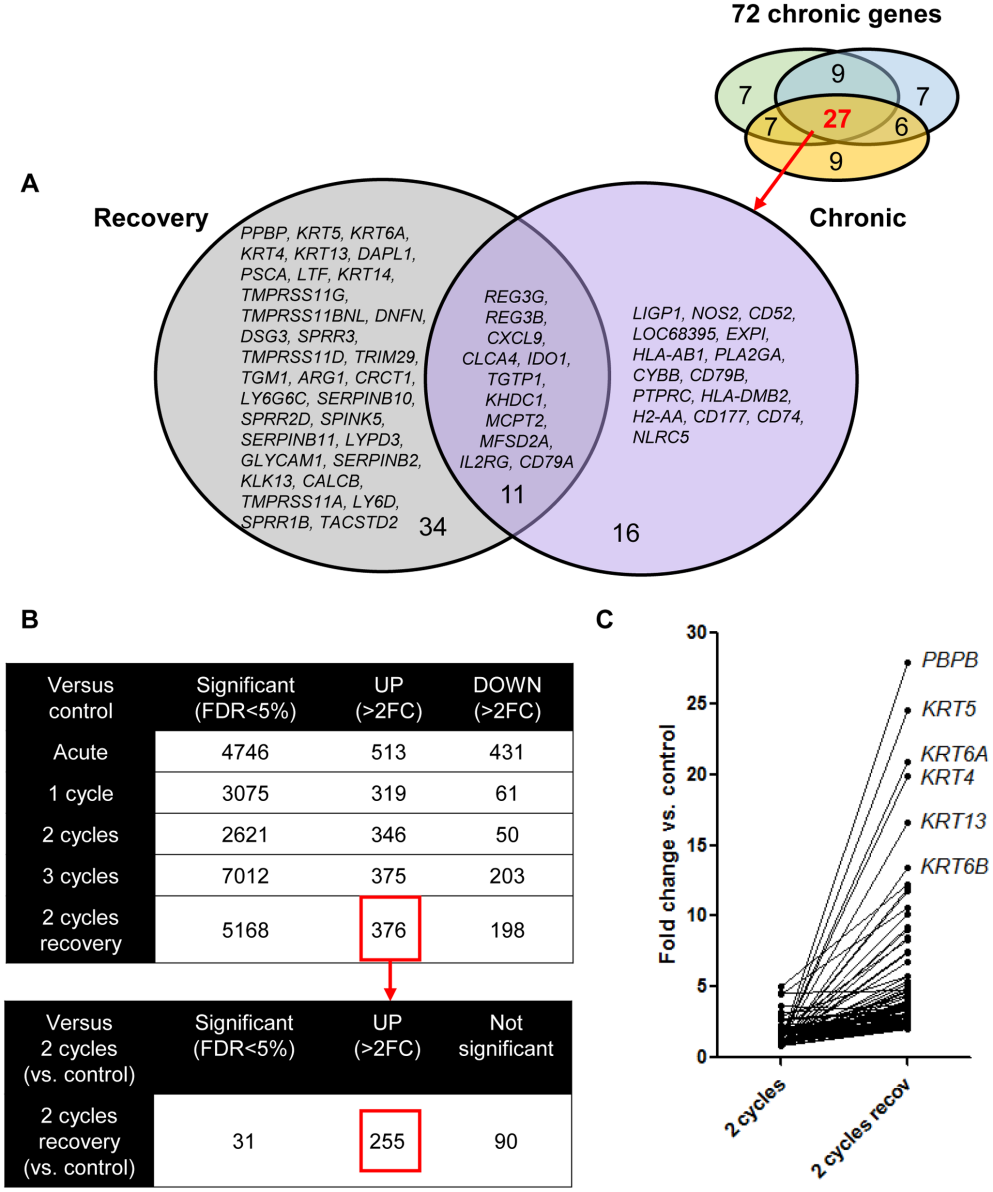
Colonic gene expression in 2 cycles DSS colitis followed by an additional recovery period of 3 weeks. (A) Venn diagram of the differentially expressed probe sets of the top 50 most upregulated genes (fold change compared to controls) after additional recovery (Table S4). These genes were compared with the 72 genes identified in chronic colitis. In total, 34 genes were uniquely upregulated in the recovery group. Only 16 genes were common with chronic colitis. Eleven out of these 16 genes were common with the 27 common upregulated genes in chronic colitis. (B) To isolate the impact of recovery on gene expression unrelated to the degree of chronicity, we identified all significantly upregulated genes after additional recovery compared to 2 cycles DSS colitis followed by immediate sacrifice. We started from all significantly upregulated genes after 2 cycles of DSS followed by additional recovery. Of these 376 genes, 255 were also upregulated after 2 cycles of DSS while 90 genes were uniquely upregulated after additional recovery (Table S5), accounting for 65 unique genes. Among these 65 genes, 8 genes coded for keratins (*KRT 4, KRT5, KRT6, KRT13, KRT14, KRT16, KRT17, KRT84*). The biological functions of the 65 genes uniquely upregulated after recovery are summarized in Table 1. (C) Individual fold change expression of the 90 unique genes significantly upregulated (fold change compared to controls) in 2 cycles DSS colitis with additional recovery vs. 2 cycles DSS colitis. The abbreviations of the individual genes and gene probe IDs are explained in the Table S4 and Table S5.

Similarly, we studied the effect of additional recovery after 2 cycles of DSS. We identified first the top 50 genes significantly upregulated in the 2-cycles group with additional recovery (Table S4) and compared these genes with the 72 significantly upregulated genes identified in chronic colitis. The made comparisons are showed in Figure 7C. In this way, 106 genes could be identified. Out of these 106 genes, 34 genes were uniquely upregulated in the recovery group and 56 genes in the chronic group. Only 16 genes were common with chronic colitis. Eleven out of these 16 genes were common with the 27 common upregulated genes in chronic colitis (*REG3G, REG3B, CXCL9, CLCA4, IDO1, TGTP1, KHDC1A, MCPT2, MFSD2A, IL2RG, CD79A)* (Figure 10A).

To isolate the impact of recovery on gene expression unrelated to the degree of chronicity, we identified all significantly upregulated genes after additional recovery compared to 2 cycles DSS colitis followed by immediate sacrifice. We started from all significantly upregulated genes after 2 cycles of DSS followed by additional recovery (Figure 10B). Of these 376 genes, 255 were also upregulated after 2 cycles of DSS while 90 genes were uniquely upregulated after additional recovery (Table S5, Figure 10C), accounting for 65 unique genes. Among these 65 genes, 8 genes coded for keratins (*KRT4, KRT5, KRT6, KRT13, KRT14, KRT16, KRT17, KRT84*) and 5 of these keratin genes were in the top 15 significantly upregulated genes after additional recovery. The biological functions of the 65 genes uniquely upregulated after recovery are summarized in Table 1. In summary, these analyses show a clear difference in colonic gene expression of the identified clusters: acute colitis and additional recovery after induction of inflammation and fibrosis both induce a typical gene expression pattern different from that in more chronic colitis and different from each other. In chronic colitis with either 1, 2 and 3 cycles of DSS, the expression pattern of the top upregulated genes is comparable, although the effect on the level of fibrosis is different: a clear induction of fibrosis in only present after 2 and 3 cycles of DSS.

### *In vivo* MRI *T*_2_ Relaxometry as a Non-invasive Assessment Tool for Discrimination of Bowel Wall Inflammation and Fibrosis

**Figure 11:**
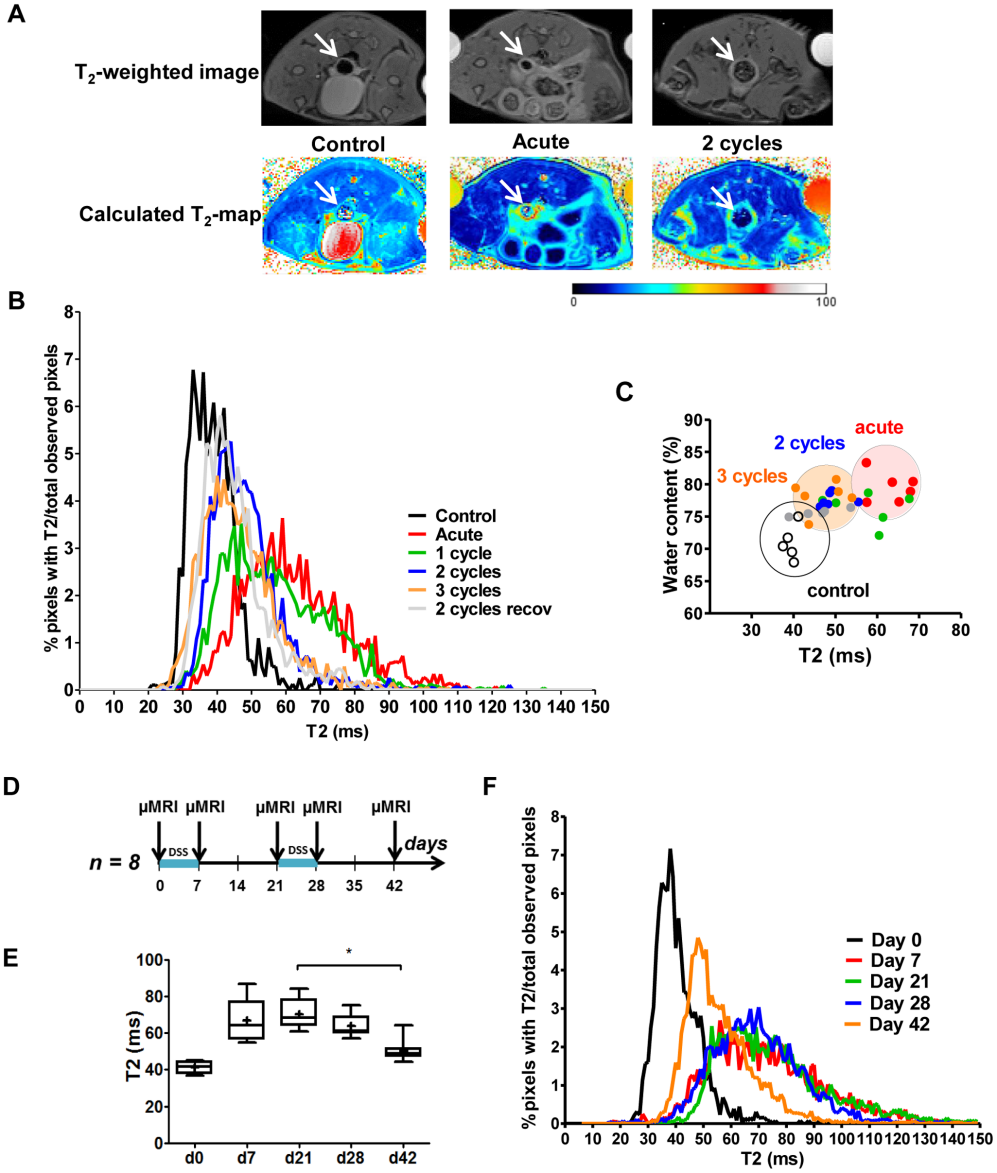
MRI of the colon. (A) Example of a *T_2_*w image and corresponding *T_2_* map of a control, acute and 2-cycles colon shows high *T_2_* in acute colon and intermediate *T_2_* after 2 cycles of DSS. (B) The *T_2_* histogram of control colon (black) shifts to higher values after 7 days of DSS (red, acute model). With more cycles of DSS, the *T_2_* map shifts back to lower values. In the 2-, and 3-cycles model, the histogram of the colon goes to an intermediate state in between the normal colon and the acute colitis. The statistical analysis of these profiles is shown in table 2. (C) Although the water content of acute colitis tissue vs. 2- and 3-cycles DSS colitis is the same, *T_2_* values are clearly different. (D) As a proof of concept, eight mice were sequentially scanned through a 2-cycles model. Mice were scanned at day 0, day 7, day 21, day 28 and day 42. (E) In this set-up, the mean *T_2_* values of the distal colon after 2 cycles (day 42) show a significant decrease compared to the 1-cycle (21 days) time point (*, p = 0.016). Data are expressed as the mean value per mouse of *T_2_* of three images, non-parametric testing. (F) The histograms show more clearly the changes in *T_2_* profiles of the different scan time points following the same trend as in the endpoint-scanning.

*In vivo* MRI *T_2_*-relaxometry was performed before sacrifice in 5–6 mice per experimental condition (Figure 11A). Analysis of the *T_2_* profiles showed that *T_2_* relaxometry was able to discriminate between all groups: in the acute phase, a clear shift was seen towards higher *T_2_* values versus normal colon. Increasing number of cycles correlated with a gradual regression of *T_2_* values to those of normal colon (Figure 11B). Analysis of the histograms showed significant differences between the groups (Table 2). The histogram of a control colon was significantly different compared to the acute group, 1-, 2- and 3-cycles DSS group. The histogram of the acute colon was significantly different compared to the 2-, 3-, and 2-cycles with additional recovery group. *T_2_* in part reflects the water content per pixel, so tissue edema is associated with a higher *T_2_*. Although the water content of acute colitis tissue versus the 2-, and 3-cycles colon was not different (p = 0.065 and 0.589 respectively), analysis of the *T_2_* profiles was able to differentiate between these conditions (Figure 11C). As a proof of concept that *T_2_* relaxometry indeed would allow *in vivo* monitoring of disease status, mice (n = 8) were sequentially scanned throughout a 2-cycles model (Figure 11D). The mean *T_2_* value after 2 cycles (day 42) showed a significant decrease compared to that after 1 cycle (21 days) (p = 0.016). *T_2_* values at day 42 also tended to be increased versus baseline levels (day 0: *T_2_* = 42±3 ms) (Figure 11E). Analysis of the *T_2_* maps showed a similar profile as observed in the previous endpoint scan in the different experimental conditions (Figure 11F).

**Table 2:** Statistical analysis of the MRI *T_2_* profiles.

**Conditions**			**p uncorrected**	**p corrected**
Control	versus	acute	<0.001	<0.015 *
		1 cycle	<0.001	<0.015 *
		2 cycles	<0.001	<0.015 *
		3 cycles	0.003	0.045 *
		2 cycles recov	0.057	ns
**Acute**	versus	1 cycle	0.028	ns
		2 cycles	<0.001	<0.015 *
		3 cycles	<0.001	<0.015 *
		2 cycles recov	<0.001	<0.015 *
**1 cycle**	versus	2 cycles	0.080	ns
		3 cycles	0.029	ns
		2 cycles recov	0.019	ns
**2 cycles**	versus	3 cycles	0.080	ns
		2 cycles recov	0.050	ns
**2 cycles recov**	versus	2 cycles recov	0.420	ns

(ns = not significant).

## Discussion

The current study aimed to characterize a murine model of progressive, transmural inflammatory colitis which more closely reflects the course of human CD. We show that repeated cycles of administration of DSS for 1 week followed by a recovery phase of 2 weeks, induce a relapsing and remitting disease course with transmural inflammation and typical connective tissue changes reflecting human CD. The gene expression profiles in chronic colitis differed from the ones associated with acute inflammation and additional recovery. Furthermore, our results show the potential of *in vivo* MRI *T*_2_ relaxometry as a non-invasive imaging tool to discriminate between acute and chronic phases of bowel wall inflammation and fibrosis.

Repeated cycles of exposure to DSS led to persistent infiltration of lymphocytes and lymphoid cell aggregates throughout all colon layers, which is a unique feature of human CD. This indicates that repeated cycles of DSS can induce persisting inflammation while the active neutrophil inflammation diminishes. More cycles of administration of DSS also resulted in a deposition of collagen I in the mucosa and submucosa and of collagen III in the muscle layer, similar to what is seen in CD and intestinal stricture formation [29]. Myofibroblasts in submucosal position, the hallmark of intestinal fibrosis, were only present in the chronic phase (i.e. after 2 or more administrations of DSS) and not in acute colitis without recovery. During additional recovery after 2 administrations of DSS, a mucosal healing response occurred with normalization of the thickness of the mucosa reflecting a high potential for mucosal restoration. However, persistent thickening of the muscularis propria was observed, also after additional recovery, which is thought to reflect mesenchymal remodeling, as seen in CD. This hypothesis is also underscored by the presence of fibroblasts and newly synthesized collagen III between the muscular fibers of the muscularis propria. These connective tissue cells may be important for the persistent inflammation [30].

Contrary to earlier reports showing a fibrogenic response after a single DSS administration [13], [14], we only observed clear fibrosis after at least 2 cycles of administration of DSS followed by 2 weeks of recovery. We also demonstrate that prolonged recovery after 2 cycles of DSS was associated with decreased fibrosis. An apparent discrepancy was noted for the degree of fibrosis in respect to the group depending on the assessment tool, MSB-staining or the hydroxyprolin assay (Figure 4B,C). This is probably due to a sampling bias. For histology, we looked at two cross-sections of the colon per mouse and assessed the amount of blue color semi quantitatively (fibrosis) in the mucosa and submucosa. However, for the hydroxyprolin assay, we measured the concentration of collagen in a piece of full wall colon, including the muscular layers. The higher level of collagen in the hydroxyprolin assay of the group with additional recovery is probably also due to the presence of remnants of collagen in the tissue.

The expression of tenascin in response to repeated cycles of DSS has not been studied in detail in animal models. The most interesting finding is the correlation of tenascin expression after repeated DSS cycles with its expression in Crohn’s colitis. As in the non-diseased human bowel wall, tenascin expression was scant in control mice. Similar to both CD and UC, the expression was increased in granulation tissue, confirming the occurrence of alterations in the ECM in association with the relapsing inflammatory process typical for IBD. The increased expression of tenascin in the submucosa underscores that this model is mimicking human CD, as the expression of tenascin in the submucosa of UC is largely similar to what is seen in the normal colon [5], [31]. In the present study, the increased expression of tenascin in acute colitis is in accordance with the role of tenascin in wound healing and migration of proliferating cells. These findings persist after 2 cycles of administration of DSS followed by recovery as actual tissue damage and repair are still ongoing. Similarly, the expression of tenascin decreased after an additional cycle or after prolonged recovery after 2 cycles since no active tissue damage is present at these later timepoints.

A strength of this paper is the use of C57BL/6 mice and the different cycles of administration of DSS followed by recovery. We studied the effect of 2 cycles of DSS and evaluated the effect of one cycle of DSS less or more. We compared this to the conventional acute DSS model and also studied the effect for additional recovery after 2 cycles of DSS. Indeed, most transgenic and conditional KO strains are available in C57BL/6 background and previously limited success has been obtained at establishing intestinal fibrosis in this strain. Recently, the use of the DSS model to study tissue remodeling has been described in a C57BL/6 Col I-GFP reporter strain [10]. In some additional papers, fibrosis has been described in the DSS model, but only by using one cycle of DSS [13], [14] or using different administrations of DSS with very short recovery periods [32]. Using the different cycles of administration of DSS followed by recovery, we were able to study on a longitudinal way the effects of additional recovery and additional administrations of DSS, showing that there is clearly a stronger induction of fibrosis with one or two additional cycles of administration of DSS followed by a 2-weeks recovery period and that only this repeated cycles model is associated with typical connective tissue changes as seen in human IBD. This offers the opportunity to compare acute and chronic repair mechanisms. In addition, our data show that with extended recovery after the last exposure connective tissue changes prevail in the absence of marked active inflammation.

Furthermore, we performed microarray to study the colonic gene expression in the different phases of inflammation and fibrosis, providing information of the differential regulation of genes during the spontaneous fibrosis recovery. This opens perspectives to study potential therapeutic targets that can reverse fibrosis. To date, to our knowledge, we are the first to show microarray data of colonic gene expression in a chronic murine model of IBD. Using the different cycles of administration of DSS followed by recovery, we were able to study colonic gene expression patterns longitudinally, and show differences between acute and chronic repair mechanisms. Unsupervised hierarchical cluster analysis based on log2 expression values of the top 50 most variable gene probe sets showed 4 different clusters: control mice, acute DSS colitis, chronic DSS colitis defined as 1-, 2- and 3-cycles DSS colitis and an additional recovery group after induction of fibrosis. After acute DSS colitis, gene expression levels, mostly of inflammation induced genes, were markedly upregulated. Although histological inflammation persists (Figure 3B), the levels of gene expression of the top 50 significantly most upregulated genes in acute DSS colitis was 3 to 9 times lower, with additional administrations of DSS followed by 2 weeks of recovery (chronic colitis). An additional recovery period after induction of fibrosis (2 cycles vs. 2 cycles with additional recovery) induces an unique gene expression profile with upregulation of specific genes, in particular keratins. The unique upregulation of keratins with additional recovery after 2 administrations of DSS is an interesting finding. Keratins are the largest subgroup of intermediate filament proteins, which are an important constituent of the cellular cytoskeleton. The specific keratin profile of a particular epithelium provides it with strength and integrity. Previously, it has been shown that keratin null mice develop spontaneous colitis [33], [34], and abnormal keratin mutations have also been shown to be associated with IBD [35]. More detailed focus on these different gene expression profiles can lead to the identification of genetic markers with a biopredictive power of inflammation and tissue repair.

On the other hand, *REG3G, REG3B, CXCL9, CLCA4* and *IDO1* are highly expressed in all conditions, although significantly higher in acute colitis compared to the more cycles of DSS administration followed by a 2-weeks recovery period (Figure 8B). Regenerating islet-derived protein 3 gamma and beta (*REG3G* and *REG3B*), antimicrobial C-type lectins, appear to be important in inflammatory diseases and intestinal injury as their expression is increased in IBD patients and in DSS models of murine colitis. Recently, it has been demonstrated that *REG3G* restricts bacterial colonization of the intestinal epithelial surface and consequently limits activation of adaptive immune responses by the microbiota [36]. Our results confirm the association of these proteins with colonic inflammation but they don’t separate acute from more chronic stages.

Research on intestinal fibrosis has been challenging in part because of a lack of suitable animal models [37]. Hence, having an appropriate animal model of chronically relapsing disease would undoubtedly add to the understanding of the biology of fibrosis [10]. The development of anti-fibrotic therapy would greatly benefit from the availability of an animal model that progresses toward chronic inflammation and so may offer the opportunity to compare acute and chronic repair mechanisms [10], which was the major goal of this study. Indeed, while long term medical therapy for IBD can influence significantly the inflammatory aspects of the disease, the connective tissue changes usually persist, leading to stricture formation, fibrosis and surgery [2]. Mucosal healing with medical therapy has been studied in some detail, but transmural lesions may be equally relevant for disease progression. Treating patients early in the disease course may prevent transmural complications such as fibrosis and fistulas, but treating at very early stages has been a major challenge in clinical practice.

To study more chronic phases of experimental colitis, non-invasive imaging tools that can be repetitively applied to the same animal would be a major asset. Here, we present for the first time the use of *in vivo* MRI *T_2_* relaxometry as a non-invasive assessment tool of inflammation and fibrosis in murine colitis. *T_2_* relaxometry was able to discern between the effects of different cycles of DSS and correlated with the observed histological changes, allowing *in vivo* monitoring of disease status. The lower *T_2_* due to the fibrosis component compared to a higher *T_2_* in case of inflammation is expected but different problems make that we decided not to state directly that we can measure fibrosis with this technique as such without a component of inflammation (tissue edema).

Tissue edema will in part be reflected by a higher water content per pixel. Since water has a higher *T_2_* than normal tissue, pixels with substantial tissue edema will be associated with an increased *T_2_*. Thus, the shift of the histogram of the colon to lower *T_2_* values with more cycles of DSS can in part be explained by a decrease of active inflammation which is prominent in the acute DSS model. However, we did not observe differences in water content of the acute colitis tissue vs. the 2-, and 3-cycles colitis tissue. Moreover, more cycles of DSS were associated with a significant increase in deposition of extracellular matrix. This suggests that the decrease in inflammation can never be fully responsible for the shift in *T_2_* in chronic DSS. The shift in *T_2_* is probably not just reflecting a lower water content due to a reduction in inflammation and is likely to be influenced by other factors such as the progressive fibrosis. The current data do not allow to completely separate both components due to the low pixel resolution of the MR images relative to the colon wall thickness in mice, the different signals of fibrosis and inflammation/tissue edema cannot be evaluated as such as both signals are present within the same MRI pixel. The relaxometry represents the global evaluation within the pixels of the bowel wall as a whole instead of regions within the wall with fibrosis or inflammation separately. In addition, the first healing phase after the acute inflammation could explain a first shift towards lower *T_2_* followed by a later further reduction in *T_2_* more caused by increasing fibrosis. Significant increases in the fibrosis score without changes in inflammation between groups would be needed to provide evidence for this hypothesis. Moreover, data indicate that strictures in human CD are most often composed of a variable range of inflammatory and fibrotic regions. However, in patients (and larger animals), due to the larger pixel density in MR enterography, it is likely that *T_2_* relaxometry algorithms can be applied to the layers of the bowel wall and to determine what proportion of the transmural disease is inflammatory. This would allow to delineate clear regions within the same image of the bowel wall of fibrosis or inflammation and to study the longitudinal changes in *T_2_* for both. Altogether, these findings suggest that *in vivo T_2_* relaxometry is a promising non-invasive assessment tool of inflammation and fibrosis of the bowel wall in colitis but more studies are necessary to reveal the main reason for the shift in *T_2_* and to determine meaningful cut offs associated with pre-dominant fibrosis or inflammation.

In summary, the murine repeated cycles of DSS colitis model opens perspectives for studying the effects of healing and fibrosis in CD. It addresses the need for more reliable animal models which mimic the chronically relapsing inflammation underlying the complications of human CD. The technical ease of the DSS model and ability to induce it in mice of common genetic background makes it an attractive model to study the connective tissue changes and intestinal fibrosis occurring in IBD. This will undoubtedly add to the understanding of the biology of intestinal wound healing and can lead to the development of more effective and more targeted therapeutic strategies for intestinal fibrosis and remodeling.

## Supporting Information

Figure S1 **Detail of the histological score of inflammation.** The score of histological inflammation, with a maximum score of 15, was calculated as the sum of following parameters: mucosal architecture change (0, none; 1, focal and mild; 2, multifocal or diffuse and mild to moderate; 3, multifocal or diffuse and severe), mononuclear cell infiltration (0, within normal limits; 1, slightly increased infiltrate in the lamina propria; 2, dense infiltrate in the lamina propria; 3, cell aggregates in the mucosa or submucosa), neutrophil infiltration (0, none; 1, in the lamina propria with or without cryptitis; 2, one or more crypt abscesses; 3, infiltration of neutrophils in the mucosa or submucosa), epithelial defects (0, none; 1, unequivocable focal erosion; 2, multifocal erosion; 3, ulceration) and goblet cell loss (0, none; 1, focal; 2, multifocal; 3, generalized). Per mouse, the mean score of two cross-sections and one longitudinal section of each of the five parameters was calculated. Data are expressed as medians with IQR. Mann-Whitney U testing (*p<0.05, **p≤0.01, ***p≤0.001).
(TIF)

Table S1 **Unsupervised hierarchical clustering of the top 50 gene probe sets with the highest variation in expression across the 30 arrays.**
(DOCX)

Table S2 **The 72 unique genes identified in the top 50 significantly upregulated genes of 1-, 2-, and 3-cycles DSS colitis (FDR<0.05, FC>2) (fold change versus controls).**
(DOCX)

Table S3 **Top 50 significantly upregulated genes in acute colitis (fold change versus controls).**
(DOCX)

Table S4 **Top 50 significantly upregulated genes in 2-cycles DSS colitis with additional recovery (fold change versus controls).**
(DOCX)

Table S5 **The 90 significantly upregulated genes uniquely upregulated after additional recovery (2 cycles of DSS administration followed by an additional recovery period compared to 2-cycles DSS colitis).**
(DOCX)
