# Supplementary figures and images for: Unique Gene Expression and MR T2 Relaxometry Patterns Define Chronic Murine Dextran Sodium Sulphate Colitis as a Model for Connective Tissue Changes in Human Crohn’s Disease
Source: PLoS One. 2013 Jul 23;8(7):e68876. doi: 10.1371/journal.pone.0068876 (PMC3720888; doi:10.1371/journal.pone.0068876)

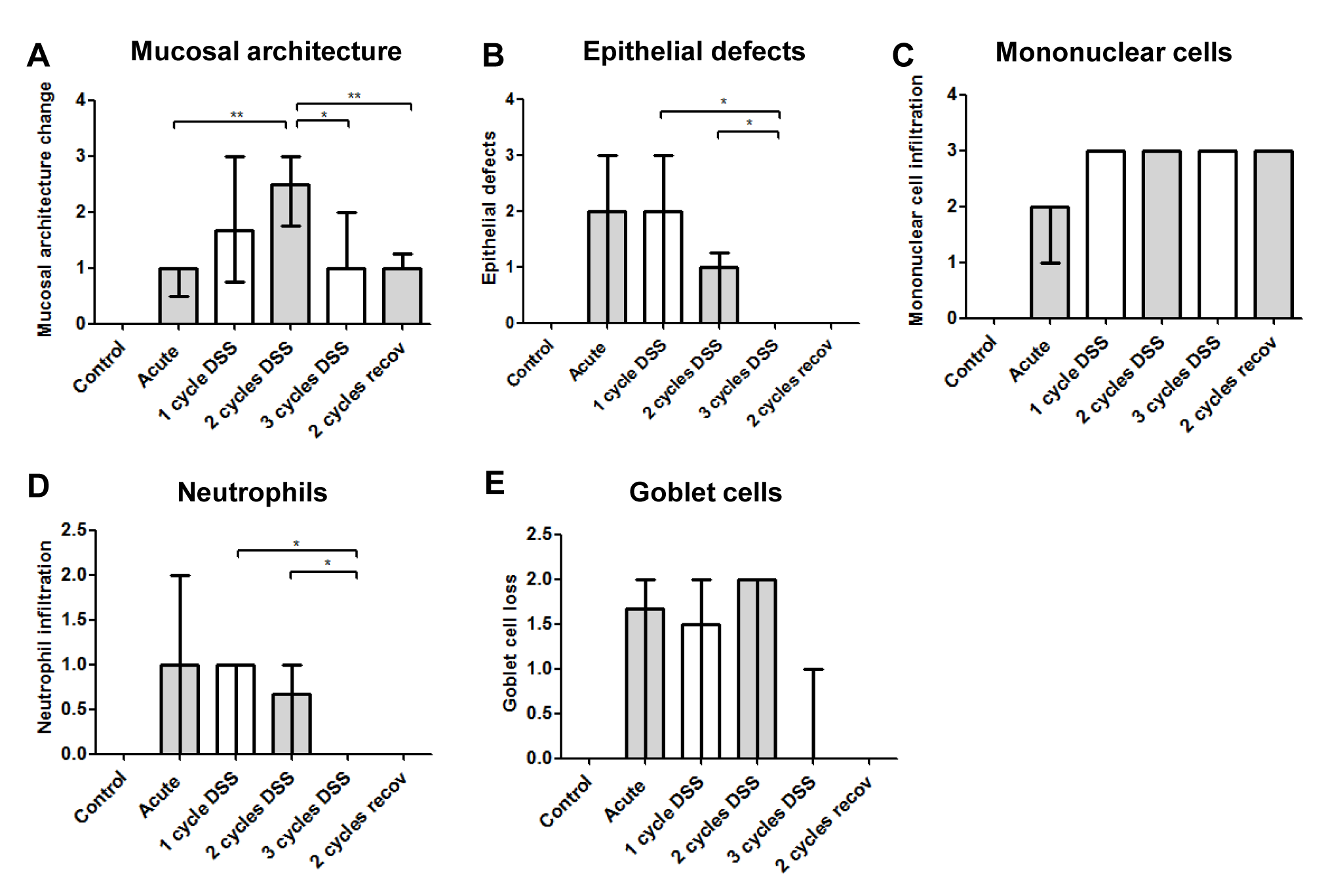

Supplement: Figure S1 — Detail of the histological score of inflammation. The score of histological inflammation, with a maximum score of 15, was calculated as the sum of following parameters: mucosal architecture change (0, none; 1, focal and mild; 2, multifocal or diffuse and mild to moderate; 3, multifocal or diffuse and severe), mononuclear cell infiltration (0, within normal limits; 1, slightly increased infiltrate in the lamina propria; 2, dense infiltrate in the lamina propria; 3, cell aggregates in the mucosa or submucosa), neutrophil infiltration (0, none; 1, in the lamina propria with or without cryptitis; 2, one or more crypt abscesses; 3, infiltration of neutrophils in the mucosa or submucosa), epithelial defects (0, none; 1, unequivocable focal erosion; 2, multifocal erosion; 3, ulceration) and goblet cell loss (0, none; 1, focal; 2, multifocal; 3, generalized). Per mouse, the mean score of two cross-sections and one longitudinal section of each of the five parameters was calculated. Data are expressed as medians with IQR. Mann-Whitney U testing (*p<0.05, **p≤0.01, ***p≤0.001). (TIF) [file pone.0068876.s001.tif]
