# Supplementary material for: Unique Gene Expression and MR T2 Relaxometry Patterns Define Chronic Murine Dextran Sodium Sulphate Colitis as a Model for Connective Tissue Changes in Human Crohn’s Disease
Source: PLoS One. 2013 Jul 23;8(7):e68876. doi: 10.1371/journal.pone.0068876 (PMC3720888; doi:10.1371/journal.pone.0068876)
Supplement: Table S1 — Unsupervised hierarchical clustering of the top 50 gene probe sets with the highest variation in expression across the 30 arrays. (DOCX) [file pone.0068876.s002.docx]

**Table S1: Unsupervised hierarchical clustering of the top 50 gene probe sets with the highest variation in expression across the 30 arrays**

| Number | Chip ID | Gene symbol |  |
| --- | --- | --- | --- |
| **1** | 10545215 | *IGK-V28* | Immunoglobulin kappa chain variable 28 |
| **2** | 10538882 | *NA* | Predicted gene 5571 |
| **3** | 10545220 | *IGK-V1* | Immunoglobulin kappa chain complex |
| **4** | 10403069 | *IGH-6* | Immunoglobulin heavy chain 6 (heavy chain IgM) |
| **5** | 10438405 | *IGL-V1* | Immunoglobulin lambda chain variable 1 |
| **6** | 10403021 | *NA* | Not assigned |
| **7** | 10538880 | *IGK-V1* | Immunoglobulin kappa chain variable 1 |
| **8** | 10538924 | *IGK-V1* | Similar to Ig kappa V – region 24B |
| **9** | 10538871 | *NA* | Predicted gene 4964 |
| **10** | 10403063 | *IGH-VJ558* | Similar to Igha protein |
| **11** | 10403034 | *IGH-VJ558* | Similar to Igha protein |
| **12** | 10545208 | *NA* | Predicted gene 189 |
| **13** | 10598085 | *NA* | ATP synthase F0 subunit 6 |
| **14** | 10545180 | *NA* | Predicted gene 10879 |
| **15** | 10545239 | *NA* | Not assigned |
| **16** | 10545249 | *NA* | Similar to Ig kappa chain V-V region MPC11 precursor |
| **17** | 10545177 | *NA* | Not assigned |
| **18** | 10438415 | *IGL-V1* | Immunoglobulin lambda chain variable 2 |
| **19** | 10403009 | *IGHG* | Immunoglobulin heavy chain (gamma polypeptide) |
| **20** | 10545569 | *REG3G* | Regenerating islet-derived 3 gamma |
| **21** | 10539179 | *REG3B* | Regenerating islet-derived 2 beta |
| **22** | 10436087 | *RETNLB* | Resistin like beta |
| **23** | 10502613 | *AI747448* | Expressed sequence AI747448 |
| **24** | 10531407 | *CXCL9* | Chemokine (C-X-C motif) ligand 9 |
| **25** | 10476042 | *TGM3* | Transglutaminase 3, E polypeptide |
| **26** | 10351959 | *1810030J14RIK* | RIKEN cDNA 1810030J14 gene |
| **27** | 10463005 | *CYP2C55* | Cytochrome P450, family 2, subfamily c, polypeptide 55 |
| **28** | 10432785 | *KRT5* | Keratin 5 |
| **29** | 10523128 | *PPBP* | Pro-plateled basic protein |
| **30** | 10432774 | *KRT6B* | Keratin 6B |
| **31** | 10391013 | *KRT13* | Keratin 13 |
| **32** | 10589703 | *LTF* | Lactotransferrin |
| **33** | 10391052 | *KRT14* | Keratin 14 |
| **34** | 10432886 | *KRT4* | Keratin 4 |
| **35** | 10432780 | *KRT6A* | Keratin 6A |
| **36** | 10424662 | *PSCA* | Prostate stem cell antigen |
| **37** | 10472235 | *DAPL1* | Death associated protein-like 1 |
| **38** | 10530986 | *9930032O22RIK* | RIKEN cDNA 9930032O22 gene |
| **39** | 10545235 | *NA* | Not assigned |
| **40** | 10545237 | *NA* | Not assigned |
| **41** | 10403036 | *IGHG* | Similar to Ig heavy chain V region 93G7 precursor |
| **42** | 10403011 | *NA* | Similar to Ig heavy chain V region IR2 precursor |
| **43** | 10403006 | *NA* | Predicted gene 7112 |
| **44** | 10451953 | *LRG1* | Leucine-rich alpha-2-glycoprotein 1 |
| **45** | 10481627 | *LCN2* | Lipocalin 2 |
| **46** | 10531415 | *CXCL10* | Chemokine (C-X-C motif) ligand 10 |
| **47** | 10583071 | *MMP3* | Matrix metallopeptidase 3 |
| **48** | 10583044 | *MMP13* | Matrix metallopeptidase 13 |
| **49** | 10563597 | *SAA3* | Serum amyloid A 3 |
| **50** | 10577655 | *IDO1* | Indoleamine 2,3-dioxygenase 1 |
