# Supplementary material for: Unique Gene Expression and MR T2 Relaxometry Patterns Define Chronic Murine Dextran Sodium Sulphate Colitis as a Model for Connective Tissue Changes in Human Crohn’s Disease
Source: PLoS One. 2013 Jul 23;8(7):e68876. doi: 10.1371/journal.pone.0068876 (PMC3720888; doi:10.1371/journal.pone.0068876)
Supplement: Table S2 — The 72 unique genes identified in the top 50 significantly upregulated genes of 1-, 2-, and 3-cycles DSS colitis (FDR<0.05, FC>2) (fold change versus controls). (DOCX) [file pone.0068876.s003.docx]

**Table S2: The 72 unique genes identified in the top 50 significantly upregulated genes of 1-, 2-, and 3-cycles DSS colitis (FDR<0.05, FC>2) (fold change versus controls)**

| Chip ID | Gene symbol | Description | Number of cycles * |
| --- | --- | --- | --- |
| 10376326 | *IGTP* | interferon gamma induced GTPase | 1 |
| 10481627 | *LCN2* | lipocalin 2 | 1 |
| 10415431 | *MCPT1* | mast cell protease 1 | 1 |
| 10583044 | *MMP13* | matrix metallopeptidase 13 (collagenase 3) | 1 |
| 10444258 | *PSMB8* | proteasome (prosome, macropain) subunit, beta type, 8 | 1 |
| 10351873 | *PYHIN1* | pyrin and HIN domain family, member 1 | 1 |
| 10563597 | *SAA3* | serum amyloid A 3 | 1 |
| 10406928 | *CD180* | CD180 molecule | 2 |
| 10567863 | *CD19* | CD19 molecule | 2 |
| 10512470 | *CD72* | CD72 molecule | 2 |
| 10379727 | *WFDC17* | WAP four-disulfide core domain 17 | 2 |
| 10487588 | *IL1A* | interleukin 1, alpha | 2 |
| 10508663 | *LAPTM5* | lysosomal protein transmembrane 5 | 2 |
| 10372648 | *LYZ2* | lysozyme 2 | 2 |
| 10379535 | *CCL8* | chemokine (C-C motif) ligand 8 | 3 |
| 10364093 | *DERL3* | derlin 3 | 3 |
| 10475448 | *DUOXA2* | dual oxidase maturation factor 2 | 3 |
| 10444229 | *H2-DMA* | major histocompatibility complex, class II, DM alpha | 3 |
| 10598087 | *ND6* | NADH dehydrogenase, subunit 6 | 3 |
| 10398039 | *SERPINA3F* | serine (or cysteine) peptidase inhibitor, clade A, member 3F | 3 |
| 10499899 | *SPRR1A* | small proline-rich protein 1A | 3 |
| 10444244 | *TAP1* | transporter 1, ATP-binding cassette, sub-family B (MDR/TAP) | 3 |
| 10490150 | *ZBP1* | Z-DNA binding protein 1 | 3 |
| 10548892 | *ARHGDIB* | Rho GDP dissociation inhibitor (GDI) beta | 12 |
| 10502335 | *BANK1* | B-cell scaffold protein with ankyrin repeats 1 | 12 |
| 10361292 | *CR2* | complement component (3d/Epstein Barr virus) receptor 2 | 12 |
| 10523359 | *CXCL13* | chemokine (C-X-C motif) ligand 13 | 12 |
| 10444306 | *H2-EB2* | histocompatibility 2, class II antigen E beta2 | 12 |
| 10444284 | *H2-OB* | major histocompatibility complex, class II, O region beta locus | 12 |
| 10390640 | *IKZF3* | IKAROS family zinc finger 3 (Aiolos) | 12 |
| 10487597 | *IL1B* | interleukin 1, beta | 12 |
| 10466172 | *MS4A1* | membrane-spanning 4-domains, subfamily A, member 1 | 12 |
| 10550476 | *CEACAM12* | carcinoembryonic antigen-related cell adhesion molecule 12 | 13 |
| 10472538 | *DHRS9* | dehydrogenase/reductase (SDR family) member 9 | 13 |
| 10375515 | *OLFR56* | olfactory receptor 56 | 13 |
| 10384044 | *MYL7* | myosin, light chain 7, regulatory | 13 |
| 10436087 | *RETNLB* | resistin like beta | 13 |
| 10398075 | *SERPINA3N* | serpin peptidase inhibitor, clade A, member 3N | 13 |
| 10377429 | *SNORD118* | small nucleolar RNA, C/D box 118 | 13 |
| 10555280 | *CHRDL2* | chordin-like 2 | 23 |
| 10538187 | *GPNMB* | glycoprotein (transmembrane) nmb | 23 |
| 10444298 | *H2-EB1* | major histocompatibility complex, class II, E beta | 23 |
| 10451953 | *LRG1* | leucine-rich alpha-2-glycoprotein 1 | 23 |
| 10404606 | *LY86* | lymphocyte antigen 86 | 23 |
| 10461721 | *MPEG1* | macrophage expressed 1 | 23 |
| 10502613 | *CLCA4* | chloride channel accessory 4 | 123 |
| 10560886 | *CD177* | CD177 molecule | 123 |
| 10517165 | *CD52* | CD52 molecule | 123 |
| 10501063 | *CD53* | CD53 molecule | 123 |
| 10456005 | *CD74* | CD74 molecule, major histocompatibility complex, class II invariant chain | 123 |
| 10551025 | *CD79A* | CD79a molecule, immunoglobulin-associated alpha | 123 |
| 10392142 | *CD79B* | CD79b molecule, immunoglobulin-associated beta | 123 |
| 10531407 | *CXCL9* | chemokine (C-X-C motif) ligand 9 | 123 |
| 10603551 | *CYBB* | cytochrome b-245, beta polypeptide | 123 |
| 10379731 | *EXPI* | extracellular proteinase inhibitor | 123 |
| 10450154 | *H2-AA* | major histocompatibility complex, class II, antigen A, alpha | 123 |
| 10444291 | *H2-AB1* | major histocompatibility complex, class II, antigen A, beta 1 | 123 |
| 10444236 | *H2-DMB2* | major histocompatibility complex, class II, locus Mb1 | 123 |
| 10444824 | *LOC68395* | histocompatibility 2, Q region locus 6-like | 123 |
| 10577655 | *IDO1* | indoleamine 2,3-dioxygenase 1 | 123 |
| 10455961 | *IIGP1* | interferon inducible GTPase 1 | 123 |
| 10606016 | *IL2RG* | interleukin 2 receptor, gamma | 123 |
| 10345077 | *KHDC1A* | KH homology domain containing 1A | 123 |
| 10415438 | *MCPT2* | mast cell protease 2 | 123 |
| 10516064 | *MFSD2A* | major facilitator superfamily domain containing 2A | 123 |
| 10574098 | *NLRC5* | NLR family, CARD domain containing 5 | 123 |
| 10379228 | *NOS2* | nitric oxide synthase 2, inducible | 123 |
| 10509584 | *PLA2G2A* | phospholipase A2, group IIA (platelets, synovial fluid) | 123 |
| 10358224 | *PTPRC* | protein tyrosine phosphatase, receptor type, C | 123 |
| 10539179 | *REG3B* | regenerating islet-derived 3 beta | 123 |
| 10545569 | *REG3G* | regenerating islet-derived 3 gamma | 123 |
| 10385518 | *TGTP1* | T cell specific GTPase 1 | 123 |

* 1 = within the top 50 most upregulated genes in 1-cycle DSS colitis (fold change versus controls), 2 = within the top 50 most upregulated genes in 2-cycles DSS colitis (fold change versus controls), 3 = within the top 50 most upregulated genes in 3-cycles DSS colitis (fold change versus controls).
