# Supplementary material for: Unique Gene Expression and MR T2 Relaxometry Patterns Define Chronic Murine Dextran Sodium Sulphate Colitis as a Model for Connective Tissue Changes in Human Crohn’s Disease
Source: PLoS One. 2013 Jul 23;8(7):e68876. doi: 10.1371/journal.pone.0068876 (PMC3720888; doi:10.1371/journal.pone.0068876)
Supplement: Table S3 — Top 50 significantly upregulated genes in acute colitis (fold change versus controls). (DOCX) [file pone.0068876.s004.docx]

**Table S3: Top 50 significantly upregulated genes in acute colitis (fold change versus controls)**

| Rank | Chip ID | Gene symbol | Description | Fold change vs. control |
| --- | --- | --- | --- | --- |
| 1 | 10539179 | *REG3B* | regenerating islet-derived 3 beta | 80.490 |
| 2 | 10545569 | *REG3G* | regenerating islet-derived 3 gamma | 77.328 |
| 3 | 10531407 | *CXCL9* | chemokine (C-X-C motif) ligand 9 | 43.630 |
| 4 | 10577655 | *IDO1* | indoleamine 2,3-dioxygenase 1 | 34.163 |
| 5 | 10563597 | *SAA3* | serum amyloid A 3 | 31.234 |
| 6 | 10502613 | *CLCA4* | chloride channel accessory 4 | 23.870 |
| 7 | 10583044 | *MMP13* | matrix metallopeptidase 13 (collagenase 3) | 23.193 |
| 8 | 10451953 | *LRG1* | leucine-rich alpha-2-glycoprotein 1 | 22.453 |
| 9 | 10481627 | *LCN2* | lipocalin 2 | 22.246 |
| 10 | 10379228 | *NOS2* | nitric oxide synthase 2, inducible | 20.108 |
| 11 | 10531415 | *CXCL10* | chemokine (C-X-C motif) ligand 10 | 18.660 |
| 12 | 10583071 | *MMP3* | matrix metallopeptidase 3 (stromelysin 1, progelatinase) | 17.224 |
| 13 | 10455961 | *IIGP1* | interferon inducible GTPase 1 | 17.174 |
| 14 | 10487597 | *IL1B* | interleukin 1, beta | 16.890 |
| 15 | 10487588 | *IL1A* | interleukin 1, alpha | 15.342 |
| 16 | 10385518 | *TGTP1* | T cell specific GTPase 1 | 14.899 |
| 17 | 10547664 | *CLEC4E* | C-type lectin domain family 4, member E | 11.877 |
| 18 | 10523120 | *CXCL5* | chemokine (C-X-C motif) ligand 5 | 11.813 |
| 19 | 10496539 | *GBP5* | guanylate binding protein 5 | 11.553 |
| 20 | 10416837 | *IRG1* | immunoresponsive 1 homolog (mouse) | 11.051 |
| 21 | 10523156 | *CXCL2* | chemokine (C-X-C motif) ligand 2 | 11.018 |
| 22 | 10436087 | *RETNLB* | resistin like beta | 10.982 |
| 23 | 10583090 | *MMP10* | matrix metallopeptidase 10 (stromelysin 2) | 10.846 |
| 24 | 10531987 | *GBP4* | guanylate binding protein 4 | 10.193 |
| 25 | 10360406 | *IFI205* | interferon activated gene 205 | 10.067 |
| 26 | 10565255 | *KIAA1199* | KIAA1199 | 9.996 |
| 27 | 10466210 | *MS4A6D* | membrane-spanning 4-domains, subfamily A, member 6D | 9.717 |
| 28 | 10398039 | *SERPINA3G* | serine (or cysteine) peptidase inhibitor, clade A, member 3G | 9.593 |
| 29 | 10516064 | *MFSD2A* | major facilitator superfamily domain containing 2A | 9.575 |
| 30 | 10389231 | *CCL3* | chemokine (C-C motif) ligand 3 | 9.512 |
| 31 | 10436100 | *RETNLG* | resistin like gamma | 9.490 |
| 32 | 10429560 | *LY6I* | lymphocyte antigen 6 complex, locus I | 9.262 |
| 33 | 10379511 | *CCL2* | chemokine (C-C motif) ligand 2 | 9.168 |
| 34 | 10581605 | *HP* | haptoglobin | 8.883 |
| 35 | 10499861 | *S100A9* | S100 calcium binding protein A9 | 8.745 |
| 36 | 10398075 | *SERPINA3N* | serpin peptidase inhibitor, clade A (alpha-1 antiproteinase, antitrypsin), member 3N | 8.203 |
| 37 | 10496592 | *GBP2* | guanylate binding protein 2 | 7.679 |
| 38 | 10493831 | *S100A8* | S100 calcium binding protein A8 | 7.669 |
| 39 | 10347291 | *CXCR2* | chemokine (C-X-C motif) receptor 2 | 7.663 |
| 40 | 10598976 | *TIMP1* | TIMP metallopeptidase inhibitor 1 | 7.123 |
| 41 | 10523175 | *EREG* | epiregulin | 7.083 |
| 42 | 10498024 | *SLC7A11* | solute carrier family 7 (anionic amino acid transporter light chain, xc- system), member 11 | 6.792 |
| 43 | 10379633 | *SLFN1* | schlafen 1 | 6.683 |
| 44 | 10603551 | *CYBB* | cytochrome b-245, beta polypeptide | 6.640 |
| 45 | 10363070 | *GP49A* | glycoprotein 49 A | 6.614 |
| 46 | 10375515 | *OLFR56* | olfactory receptor 56 | 6.464 |
| 47 | 10389207 | *CCL5* | chemokine (C-C motif) ligand 5 | 6.438 |
| 48 | 10574102 | *NLRC* | NLR family, CARD domain containing 5 | 6.298 |
| 49 | 10461594 | *MS4A4C* | membrane-spanning 4-domains, subfamily A, member 4C | 6.250 |
| 50 | 10376326 | *IGTP* | interferon gamma induced GTPase | 5.972 |
