# Supplementary material for: Unique Gene Expression and MR T2 Relaxometry Patterns Define Chronic Murine Dextran Sodium Sulphate Colitis as a Model for Connective Tissue Changes in Human Crohn’s Disease
Source: PLoS One. 2013 Jul 23;8(7):e68876. doi: 10.1371/journal.pone.0068876 (PMC3720888; doi:10.1371/journal.pone.0068876)
Supplement: Table S4 — Top 50 significantly upregulated genes in 2-cycles DSS colitis with additional recovery (fold change versus controls). (DOCX) [file pone.0068876.s005.docx]

**Table S4: Top 50 significantly upregulated genes in 2-cycles DSS colitis with additional recovery (fold change versus controls).**

| Rank | Chip ID | Gene symbol | Description | Fold change vs. control |
| --- | --- | --- | --- | --- |
| 1 | 10523128 | *PPBP* | pro-platelet basic protein (chemokine (C-X-C motif) ligand 7) | 27.950 |
| 2 | 10545569 | *REG3G* | regenerating islet-derived 3 gamma | 26.697 |
| 3 | 10539179 | *REG3B* | regenerating islet-derived 3 beta | 24.598 |
| 4 | 10432785 | *KRT5* | keratin 5 | 24.545 |
| 5 | 10432780 | *KRT6A* | keratin 6A | 20.915 |
| 6 | 10432886 | *KRT4* | keratin 4 | 19.897 |
| 7 | 10391013 | *KRT13* | keratin 13 | 16.576 |
| 8 | 10472235 | *DAPL1* | death associated protein-like 1 | 11.926 |
| 9 | 10424662 | *PSCA* | prostate stem cell antigen | 11.802 |
| 10 | 10589703 | *LTF* | lactotransferrin | 10.742 |
| 11 | 10436087 | *RETNLB* | resistin like beta | 10.571 |
| 12 | 10391052 | *KRT14* | keratin 14 | 10.164 |
| 13 | 10530986 | *TMPRSS11G* | transmembrane protease, serine 11g | 9.234 |
| 14 | 10531009 | *TMPRSS11BNL* | transmembrane protease, serine 11b N terminal like | 9.021 |
| 15 | 10561025 | *CNFN* | cornifelin | 8.529 |
| 16 | 10499899 | *SPRR1A* | small proline-rich protein 1A | 8.332 |
| 17 | 10454154 | *DSG3* | desmoglein 3 | 7.451 |
| 18 | 10499896 | *SPRR3* | small proline-rich protein 3 | 7.438 |
| 19 | 10530960 | *TMPRSS11D* | transmembrane protease, serine 11D | 7.402 |
| 20 | 10531407 | *CXCL9* | chemokine (C-X-C motif) ligand 9 | 7.361 |
| 21 | 10584604 | *TRIM29* | tripartite motif containing 29 | 7.352 |
| 22 | 10420114 | *TGM1* | transglutaminase 1 (K polypeptide epidermal type I, protein-glutamine-gamma-glutamyltransferase) | 7.234 |
| 23 | 10345077 | *KHDC1A* | KH homology domain containing 1A | 7.232 |
| 24 | 10502613 | *CLCA4* | chloride channel accessory 4 | 7.197 |
| 25 | 10368343 | *ARG1* | arginase, liver | 6.769 |
| 26 | 10499952 | *CRCT1* | cysteine-rich C-terminal 1 | 5.722 |
| 27 | 10444674 | *LY6G6C* | lymphocyte antigen 6 complex, locus G6C | 5.436 |
| 28 | 10349166 | *SERPINB10* | serpin peptidase inhibitor, clade B (ovalbumin), member 10 | 5.240 |
| 29 | 10577655 | *IDO1* | indoleamine 2,3-dioxygenase 1 | 5.147 |
| 30 | 10493864 | *SPRR2D* | small proline-rich protein 2D | 4.737 |
| 31 | 10455395 | *SPINK5* | serine peptidase inhibitor, Kazal type 5 | 4.712 |
| 32 | 10349138 | *SERPINB11* | serpin peptidase inhibitor, clade B (ovalbumin), member 11 (gene/pseudogene) | 4.644 |
| 33 | 10550980 | *LYPD3* | LY6/PLAUR domain containing 3 | 4.502 |
| 34 | 10585699 | *FABP5* | fatty acid binding protein 5 (psoriasis-associated) | 4.388 |
| 35 | 10538187 | *GPNMB* | glycoprotein (transmembrane) nmb | 4.379 |
| 36 | 10433172 | *GLYCAM1* | glycosylation dependent cell adhesion molecule 1 (pseudogene) | 4.350 |
| 37 | 10349157 | *SERPINB2* | serpin peptidase inhibitor, clade B (ovalbumin), member 2 | 4.260 |
| 38 | 10552469 | *KLK13* | kallikrein-related peptidase 13 | 4.033 |
| 39 | 10415438 | *MCPT2* | mast cell protease 2 | 4.023 |
| 40 | 10516064 | *MFSD2A* | major facilitator superfamily domain containing 2A | 4.013 |
| 41 | 10556546 | *CALCB* | calcitonin-related polypeptide, beta | 3.967 |
| 42 | 10530974 | *TMPRSS11A* | transmembrane protease, serine 11A | 3.888 |
| 43 | 10385518 | *TGTP1* | T cell specific GTPase 1 | 3.878 |
| 44 | 10429520 | *LY6D* | lymphocyte antigen 6 complex, locus D | 3.805 |
| 45 | 10384044 | *MYL7* | myosin, light chain 7, regulatory | 3.801 |
| 46 | 10551025 | *CD79A* | CD79a molecule, immunoglobulin-associated alpha | 3.794 |
| 47 | 10499891 | *SPRR1B* | small proline-rich protein 1B | 3.779 |
| 48 | 10472538 | *DHRS9* | dehydrogenase/reductase (SDR family) member 9 | 3.709 |
| 49 | 10545168 | *TACSTD2* | tumor-associated calcium signal transducer 2 | 3.685 |
| 50 | 10606016 | *IL2RG* | interleukin 2 receptor, gamma | 3.681 |
