# Supplementary material for: Unique Gene Expression and MR T2 Relaxometry Patterns Define Chronic Murine Dextran Sodium Sulphate Colitis as a Model for Connective Tissue Changes in Human Crohn’s Disease
Source: PLoS One. 2013 Jul 23;8(7):e68876. doi: 10.1371/journal.pone.0068876 (PMC3720888; doi:10.1371/journal.pone.0068876)
Supplement: Table S5 — The 90 significantly upregulated genes uniquely upregulated after additional recovery (2 cycles of DSS administration followed by an additional recovery period compared to 2-cycles DSS colitis). (DOCX) [file pone.0068876.s006.docx]

**Table S5: The 90 significantly upregulated genes uniquely upregulated after additional recovery (2 cycles of DSS administration followed by an additional recovery period compared to 2-cycles DSS colitis).**

| Rank | Chip ID | Gene symbol | Description | | Fold change vs. control | |
| --- | --- | --- | --- | --- | --- | --- |
| 1 | 10523128 | *PPBP* | | pro-platelet basic protein (chemokine (C-X-C motif) ligand 7) | | 27.950 |
| 2 | 10432785 | *KRT5* | | keratin 5 | | 24.545 |
| 3 | 10432780 | *KRT6A* | | keratin 6A | | 20.915 |
| 4 | 10432886 | *KRT4* | | keratin 4 | | 19.897 |
| 5 | 10391013 | *KRT13* | | keratin 13 | | 16.576 |
| 6 | 10432774 | *KRT6B* | | keratin 6B | | 13.429 |
| 7 | 10598085 | *ATP6* | | ATP synthase F0 subunit 6 | | 12.200 |
| 8 | 10472235 | *DAPL1* | | death associated protein-like 1 | | 11.926 |
| 9 | 10424662 | *PSCA* | | prostate stem cell antigen | | 11.802 |
| 10 | 10436087 | *RETNLB* | | resistin like beta | | 10.571 |
| 11 | 10391052 | *KRT14* | | keratin 14 | | 10.164 |
| 12 | 10530986 | *TMPRSS11G* | | transmembrane protease, serine 11g | | 9.234 |
| 13 | 10531009 | *TMPRSS11BNL* | | transmembrane protease, serine 11b N terminal like | | 9.021 |
| 14 | 10561025 | *CNFN* | | cornifelin | | 8.529 |
| 15 | 10499899 | *SPRR1A* | | small proline-rich protein 1A | | 8.332 |
| 16 | 10454154 | *DSG3* | | desmoglein 3 | | 7.451 |
| 17 | 10499896 | *SPRR3* | | small proline-rich protein 3 | | 7.438 |
| 18 | 10530960 | *TMPRSS11D* | | transmembrane protease, serine 11D | | 7.402 |
| 19 | 10368343 | *ARG1* | | arginase, liver | | 6.769 |
| 20 | 10339151 | *NA* | | Not assigned (intronic normalization control) | | 5.792 |
| 21 | 10499952 | *CRCT1* | | cysteine-rich C-terminal 1 | | 5.722 |
| 22 | 10444674 | *LY6G6C* | | lymphocyte antigen 6 complex, locus G6C | | 5.436 |
| 23 | 10349166 | *SERPINB10* | | serpin peptidase inhibitor, clade B (ovalbumin), member 10 | | 5.240 |
| 24 | 10484520 | *4833423E24RIK* | | RIKEN cDNA 4833423E24 gene | | 4.976 |
| 25 | 10403036 | *NA* | | Not assigned | | 4.790 |
| 26 | 10493864 | *SPRR2D* | | small proline-rich protein 2D | | 4.737 |
| 27 | 10455395 | *SPINK5* | | serine peptidase inhibitor, Kazal type 5 | | 4.712 |
| 28 | 10349138 | *SERPINB11* | | serpin peptidase inhibitor, clade B (ovalbumin), member 11 (gene/pseudogene) | | 4.644 |
| 29 | 10550980 | *LYPD3* | | LY6/PLAUR domain containing 3 | | 4.502 |
| 30 | 10433172 | *GLYCAM1* | | glycosylation dependent cell adhesion molecule 1 (pseudogene) | | 4.350 |
| 31 | 10349157 | *SERPINB2* | | serpin peptidase inhibitor, clade B (ovalbumin), member 2 | | 4.260 |
| 32 | 10552469 | *KLK13* | | kallikrein-related peptidase 13 | | 4.033 |
| 33 | 10556546 | *CALCB* | | calcitonin-related polypeptide, beta | | 3.967 |
| 34 | 10530974 | *TMPRSS11A* | | transmembrane protease, serine 11A | | 3.888 |
| 35 | 10499891 | *SPRR1B* | | small proline-rich protein 1B | | 3.779 |
| 36 | 10472538 | *DHRS9* | | dehydrogenase/reductase (SDR family) member 9 | | 3.709 |
| 37 | 10545168 | *TACSTD2* | | tumor-associated calcium signal transducer 2 | | 3.685 |
| 38 | 10442381 | *PRSS27* | | protease, serine 27 | | 3.623 |
| 39 | 10407416 | *CALML3* | | calmodulin-like 3 | | 3.614 |
| 40 | 10457669 | *DSC3* | | desmocollin 3 | | 3.500 |
| 41 | 10598087 | *ND6* | | NADH dehydrogenase, subunit 6 | | 3.403 |
| 42 | 10545200 | *NA* | | Not assigned | | 3.368 |
| 43 | 10355343 | *ABCA12* | | ATP-binding cassette, subfamily A, (ABC1), member 12 | | 3.363 |
| 44 | 10538878 | *NA* | | Not assigned | | 3.329 |
| 45 | 10537880 | *NA* | | Not assigned | | 3.254 |
| 46 | 10563933 | *NA* | | Not assigned | | 3.127 |
| 47 | 10450920 | *AY036118* | | ETS-related transcription factor ERF (Erf1) | | 3.035 |
| 48 | 10391066 | *KRT17* | | keratin 17 | | 3.032 |
| 49 | 10339713 | *NA* | | Not assigned (Intronic normalization control) | | 2.963 |
| 50 | 10454113 | *DSG1A* | | Desmoglein 1 alpha | | 2.959 |
| 51 | 10339066 | *NA* | | Not assigned (Intronic normalization control) | | 2.951 |
| 52 | 10523120 | *CXCL5* | | chemokine (C-X-C motif) ligand 5 | | 2.883 |
| 53 | 10550131 | *PLA2G4C* | | phospholipase A2, group IVC (cytosolic, calcium-independent) | | 2.784 |
| 54 | 10449807 | *EPHX3* | | epoxide hydrolase 3 | | 2.772 |
| 55 | 10493108 | *CRABP2* | | cellular retinoic acid binding protein 2 | | 2.655 |
| 56 | 10341131 | *NA* | | Not assigned (Intronic normalization control) | | 2.649 |
| 57 | 10493834 | *PGLYRP4* | | peptidoglycan recognition protein 4 | | 2.638 |
| 58 | 10469786 | *IL1F9* | | interleukin 36, gamma | | 2.624 |
| 59 | 10391061 | *KRT16* | | keratin 16 | | 2.614 |
| 60 | 10493979 | *RPTN* | | repetin | | 2.589 |
| 61 | 10339837 | *NA* | | Not assigned (Intronic normalization control) | | 2.573 |
| 62 | 10564417 | *ALDH1A3* | | aldehyde dehydrogenase 1 family, member A3 | | 2.571 |
| 63 | 10339985 | *NA* | | Not assigned (Intronic normalization control) | | 2.544 |
| 64 | 10522411 | *CWH43* | | cell wall biogenesis 43 C-terminal homolog (S. cerevisiae) | | 2.484 |
| 65 | 10339923 | *NA* | | Not assigned (Intronic normalization control) | | 2.472 |
| 66 | 10531022 | *TMPRSS11E* | | transmembrane protease, serine 11E | | 2.470 |
| 67 | 10435501 | *STFA1* | | stefin A1 | | 2.467 |
| 68 | 10545182 | *NA* | | Not assigned | | 2.434 |
| 69 | 10507076 | *SKINT3* | | selection and upkeep of intraepithelial T cells 6 | | 2.383 |
| 70 | 10453734 | *NA* | | Not assigned | | 2.362 |
| 71 | 10502638 | *CLCA5* | | chloride channel calcium activated 5 | | 2.354 |
| 72 | 10342785 | *NA* | | Not assigned (Intronic normalization control) | | 2.319 |
| 73 | 10586591 | *CAR12* | | carbonic anhydrase XII | | 2.296 |
| 74 | 10554034 | *LASS3* | | ceramide synthase 3 | | 2.280 |
| 75 | 10341152 | *NA* | | Not assigned (Intronic normalization control) | | 2.275 |
| 76 | 10566205 | *DUB2A* | | ubiquitin specific peptidase 17-like family member 4 | | 2.261 |
| 77 | 10338194 | *NA* | | Not assigned (Intronic normalization control) | | 2.236 |
| 78 | 10339212 | *NA* | | Not assigned (Intronic normalization control) | | 2.216 |
| 79 | 10432767 | *GM5478* | | predicted pseudogene 5478 | | 2.205 |
| 80 | 10340783 | *NA* | | Not assigned (Intronic normalization control) | | 2.185 |
| 81 | 10343794 | *NA* | | Not assigned (Intronic normalization control) | | 2.173 |
| 82 | 10466606 | *ANXA1* | | annexin A1 | | 2.154 |
| 83 | 10357077 | *SERPINB3D* | | serpin peptidase inhibitor, clade B (ovalbumin), member 3D | | 2.065 |
| 84 | 10342326 | *NA* | | Not assigned (Intronic normalization control) | | 2.050 |
| 85 | 10542355 | *EMP1* | | epithelial membrane protein 1 | | 2.048 |
| 86 | 10580678 | *CES1* | | carboxylesterase 1 | | 2.037 |
| 87 | 10485405 | *CD44* | | CD44 molecule (Indian blood group) | | 2.033 |
| 88 | 10339978 | *NA* | | Not assigned (Intronic normalization control) | | 2.027 |
| 89 | 10450525 | *GM9573* | | mucin 22 | | 2.008 |
| 90 | 10432711 | *KRT84* | | keratin 84 | | 2.007 |
